# MMP9^High^ Neutrophils are Critical Mediators of Neutrophil Extracellular Traps Formation and Myocardial Ischemia/Reperfusion Injury

**DOI:** 10.1002/advs.202415205

**Published:** 2025-03-28

**Authors:** Shiyu Hu, Feng Zhang, Jingpu Wang, Jian Zhang, Chenguang Li, Yang Lyu, Yiwen Wang, Rong Huang, Yang Gao, Hongbo Yang, Juying Qian, Wenwen Tang, Jiatian Cao, Junbo Ge

**Affiliations:** ^1^ Department of Cardiology Shanghai Institute of Cardiovascular Diseases Zhongshan Hospital Fudan University Shanghai 200032 China; ^2^ National Clinical Research Center for Interventional Medicine Shanghai 200032 China; ^3^ State Key Laboratory of Cardiovascular Diseases Zhongshan Hospital Fudan University Shanghai 200032 China; ^4^ NHC Key Laboratory of Ischemic Heart Diseases Shanghai 200032 China; ^5^ Key Laboratory of Viral Heart Diseases Chinese Academy of Medical Sciences Shanghai 200032 China; ^6^ Department of Cardiology Shanghai Fifth People's Hospital Fudan University Shanghai 200240 China; ^7^ Vascular Biology and Therapeutics Program Department of Pharmacology Yale University School of Medicine New Haven CT 06520 USA

**Keywords:** cystatin F (CST7), myocardial ischemia/reperfusion injury, neutrophil extracellular traps, single‐cell RNA sequencing, SPI1

## Abstract

Neutrophil extracellular traps (NETs) are increasingly recognized as pivotal players and potential therapeutic targets in neutrophil‐mediated reperfusion injury. Despite their importance, the effects and variability of circulating neutrophils in relation to NET formation during myocardial ischemia/reperfusion injury (MI/RI) remain inadequately characterized. In this study, single‐cell transcriptomes of neutrophils isolated from the blood of healthy donors and MI/RI patients are analyzed. The results reveal that MI/RI neutrophils transition from a high IFIT1 expression profile into four distinct states, two of which exhibit elevated MMP9 transcription. These MMP9^High^ neutrophil subpopulations are instrumental in the NET formation and correlate positively with the severity of MI/RI. Further investigation identifies the transcription factor SPI1 as a key regulator of this transition, acting through modulation of CST7 expression. Targeting SPI1 or CST7 significantly reduces the prevalence of MMP9^High^ neutrophils and NET formation, resulting in improved MI/RI outcomes. These findings offer new insights into neutrophil heterogeneity and pinpoint a specific subset critical for NET formation, underscoring their potential as diagnostic biomarkers and therapeutic targets for MI/RI management.

## Graphical abstract

During a heart attack, neutrophils rapidly activate and differentiate into a specific subset characterized by high levels of MMP9, a tissue‐damaging enzyme, via the SPI1/CST7 pathway. These neutrophils form harmful net‐like structures (Neutrophil extracellular traps, NETs) that exacerbate heart injury. Inhibiting SPI1/CST7 or blocking NET formation offers a promising approach to reducing damage and improving outcomes.

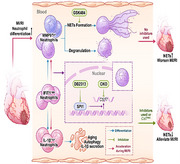

## 1 Introduction

Myocardial ischemia/reperfusion injury (MI/RI) remains a significant clinical challenge in the treatment of acute myocardial infarction (AMI) despite advances in reperfusion therapies.^[^ ^1^, ^2^ ^]^ While restoring blood flow to the ischemic myocardium is necessary for tissue survival, it paradoxically worsens injury through complex mechanisms involving inflammation, oxidative stress, and cell death.^[^ ^3^ ^]^ Neutrophils have emerged as critical mediators of tissue damage and inflammation among the various cellular and molecular players in MI/RI.

Neutrophils, as the first responders of the innate immune system, play a crucial role in the acute inflammatory response following MI/RI.^[^ ^4^ ^]^ They rapidly infiltrate the injured myocardium and release various pro‐inflammatory mediators, reactive oxygen species (ROS), and proteolytic enzymes that contribute to tissue damage.^[^ ^5^ ^]^ One of the most intriguing discoveries in neutrophil biology has been the identification of neutrophil extracellular traps (NETs).^[^ ^6^, ^7^, ^8^ ^]^ These web‐like structures, composed of DNA, histones, and granular proteins, are released by neutrophils in response to various stimuli. Recent studies have highlighted NETs' significant role in MI/RI. NETs contain many granular proteins, which can cause tissue damage through direct cytotoxic effects on cardiac tissues. The web‐like structures can also trap platelets and neutrophils, leading to thrombosis and microcirculation disturbance. The cytokines and granular proteins in NETs can amplify inflammatory responses and aggravate coagulation.^[^ ^9^, ^10^ ^]^ Our previous studies demonstrated that ALDH2 or Hdc deficiency exacerbates MI/RI by promoting NET‐related pathways.^[^ ^11^, ^12^ ^]^

Due to their heterogeneity arising from differentiation, maturation, and migration‐induced responses,^[^ ^13^, ^14^, ^15^ ^]^ neutrophils exhibit versatile and sometimes controversial roles in tissue injury and healing, particularly in MI/RI.^[^ ^16^, ^17^ ^]^ Broad treatments targeting neutrophils have not been effective in MI/RI due to the reduction of neutrophils' healing functions, such as promoting angiogenesis.^[^ ^18^ ^]^ The plasticity of neutrophil phenotypes and the redundancy in inflammatory pathways necessitate a nuanced approach to intervention. Therefore, identifying specific neutrophil subpopulations, particularly those involved in NET formation, is crucial for advancing the understanding of MI/RI biology and treatment. Recent evidence supports the existence of distinct neutrophil subsets with diverse roles in infection and inflammation, influencing the immune response to various pathogens.^[^ ^19^, ^20^ ^]^ Several studies have focused on classifying neutrophils in mice with myocardial infarction (MI) through single‐cell RNA sequencing (scRNA‐seq), identifying a new cluster of neutrophils marked by SiglecF with significant roles in the infarcted myocardium.^[^ ^21^, ^22^, ^23^ ^]^ Another study also used single‐cell RNA sequencing to analyze the anti‐inflammatory effects of YM1^High^ neutrophils in mouse MI/RI hearts.^[^ ^24^ ^]^ However, the pro‐inflammatory neutrophil subpopulations in MI/RI, especially in human samples, have not been well elucidated.

This study aimed to address existing knowledge gaps by examining the characteristics and functional properties of neutrophil subsets and NET formation within the context of MI/RI. Acknowledging the translational differences between human and mouse models, we conducted scRNA‐seq on neutrophils from MI patients 12 h post‐percutaneous coronary intervention (PCI) and healthy donors. Our findings highlighted MMP9^High^ neutrophils as primary contributors to NET formation and cardiac injury, emphasizing the complexity of neutrophil heterogeneity and their specialized roles in immune responses. We discovered that SPI1 was a key transcription factor regulating CST7 expression, which drove MMP9^High^ neutrophils  formation.

## 2 Result

### 2.1 Heterogeneity of Circulating Neutrophils in Human Blood of MI/RI Patients

**Figure 1:**
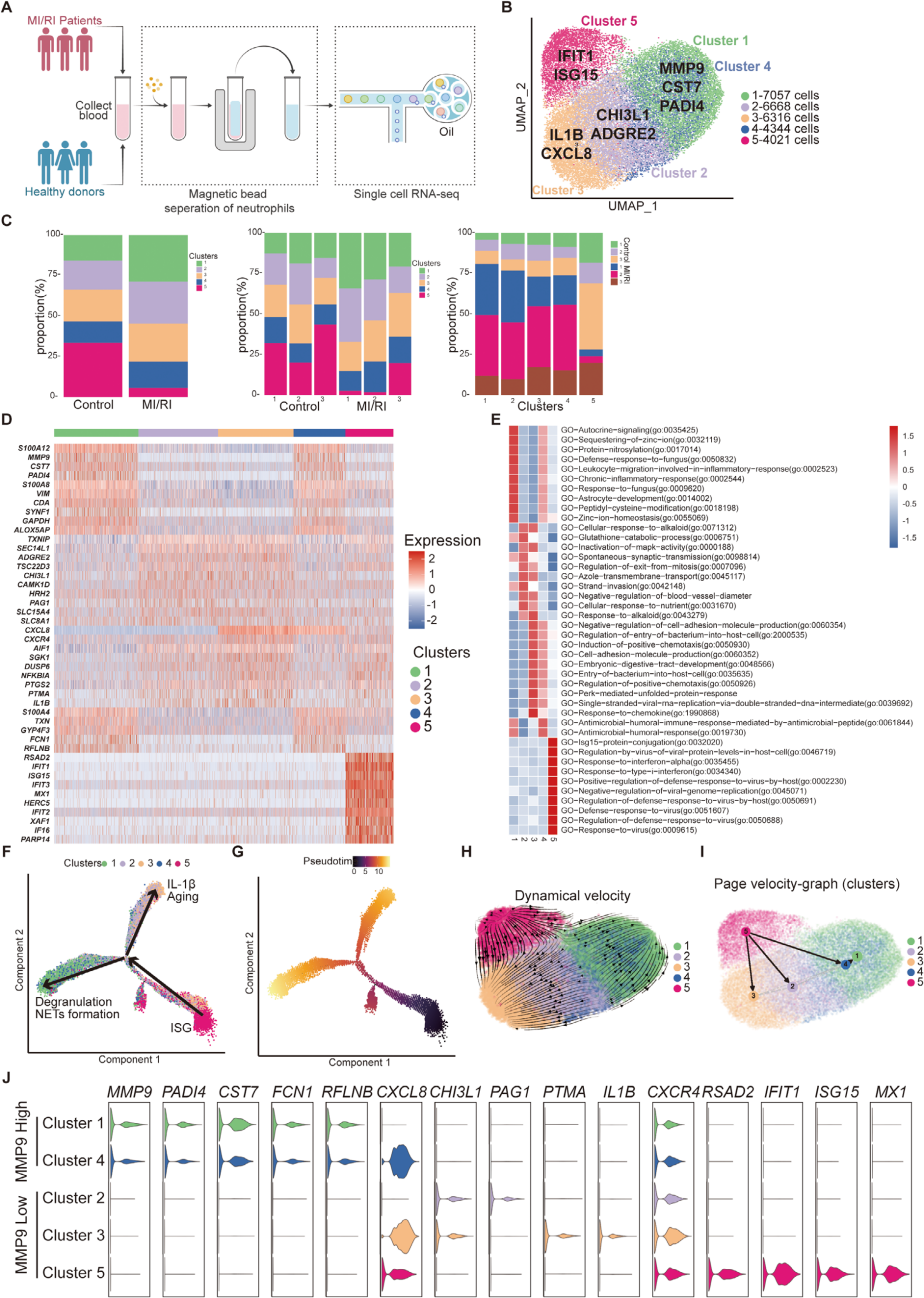
ScRNA‐seq analysis of neutrophils from the blood of MI/RI patients and healthy donors and neutrophil differentiation trajectories analysis. A) Flowchart of the experimental design. B) UMAP representation of gene expression data in Clusters 1–5 with Seurat cluster assignment projected onto the UMAP plot. C) Proportion of each cluster among total neutrophils grouped by group (left), sample (middle), and cluster (right), respectively. D) Heatmap of the top 10 marker genes (sorted by Log_2_ fold change; Bonferroni‐corrected *p *< 0.05, Student's *t*‐test) associated with the 5 neutrophil clusters (scaled average expression). E) Gene Ontology analysis of DEGs for each of the 5 clusters. Selected Gene Ontology terms with Benjamini–Hochberg‐corrected *p *< 0.05 (one‐sided Fisher's exact test) are shown. F) Monocle trajectories of neutrophils colored by cluster (differentiation direction and cluster characteristic were added additionally after the following analysis). Each dot represents a single cell. G) Pseudotime ordering of neutrophils in Monocle split according to the neutrophil cluster. H) Velocity analysis revealing the origin and inter‐relationship of neutrophil subpopulations. Velocity fields were projected onto the UMAP plot. I) Page velocity analysis graph. J) Violin plots showing characteristic gene expression in MMP9^High^ cluster and MMP9^Low^ clusters.

As the studies in both mice^[^ ^25^ ^]^ and humans^[^ ^26^ ^]^ show that neutrophils reach their peak within 24 h after MI/RI, we collected blood from 3 patients with MI 12 h after PCI and blood from 3 healthy donors. The clinical features of these subjects were detailed in Table S1 (Supporting Information). Neutrophils were separated using magnetic beads, and their purity and viability were confirmed (Figure S1A, Supporting Information). Subsequently, these neutrophils underwent 10x Genomics single‐cell RNA sequencing (scRNA‐seq), as outlined in the experimental design (**Figure** **1**A). Following rigorous gene quality control and removal of low‐quality cells, we obtained 32 291 cells with 827 genes per cell (Figure S1B, Supporting Information; detailed in Table S2, Supporting Information). Annotation of cell types and analysis of specific neutrophil markers (*FCGR3B* for human neutrophils; *KIT* and *CD34* for granulocyte‐monocyte progenitor cells)^[^ ^27^ ^]^ confirmed our cells were peripheral blood neutrophils (Figure S1C–F, Supporting Information).

After reducing the batch effects, neutrophils were classified into 5 clusters using unsupervised clustering analysis and visualized with uniform manifold approximation and projection (UMAP) (Figure 1B). Clusters 1–4 were predominant subsets in MI/RI patients, while Cluster 5 was the most abundant subset detected in healthy donors (Figure 1C; Figures S2A, and S3A, Supporting Information). The expression levels of several marker genes varied significantly among these clusters (Figure 1D; Figure S3B, Supporting Information; detailed in Table S3, Supporting Information). Specifically, Cluster 1 (*MMP9*, *CST7*, *PADI4*, *SYNE1*) and Cluster 4 (*MMP9*, *CXCL8*, *FCN1*, *PADI4*) shared many marker genes, and they were typical degranulation groups. Cluster 2 (*CHI3L1*, *ADGRE2*, *PAG1*, *SCLT1*) likely responded to bacterial presence. Cluster 3 (*CXCR4*, *PTMA*, *SGK1*, *IL1B*) showed genes associated with IL‐1β secretion and autophagy. Cluster 5 (*RSAD2*, *IFIT1*, *ISG15*, *HERC5*) was characteristic of the expression of a set of interferon‐stimulated genes (ISGs), which was consistent with the G5b subpopulation identified in the previous study.^[^ ^28^ ^]^ Gene ontology analysis of these differentially expressed genes (DEGs) corroborated the functional identities of each cluster (Figure 1E; Figure S3C, Supporting Information).

In the overall comparison of DEGs between MI/RI and the control group, upregulated genes in MI/RI patients were dispersed across Clusters 1–4, whereas downregulated genes were predominantly ISG genes (Figure S2B, Supporting Information; detailed in Table S4, Supporting Information). Gene ontology analysis illustrated an upregulation of neutrophil degranulation and a downregulation of the type I interferon signaling pathway (Figures S2C and S3D, Supporting Information), reconciling with the observed gene expression differences and cluster features.

### 2.2 Compositional Analysis of Neutrophil Heterogeneity and Functional Differentiation in MI/RI

To elucidate the relationships among the identified neutrophil clusters, Monocle was utilized to predict potential differentiation trajectories in pseudo‐time (Figure 1F; Figure S2D, Supporting Information). According to the expression of function‐related genes, we utilized the AddModuleScore to evaluate granulation, maturation, and aging within each cluster (Scoring markers detailed in Table S5, Supporting Information). Clusters 1 & 4 exhibited higher granulation scores (Figure S2E, Supporting Information) and elevated expression levels of granulation marker genes (Figure S2F, Supporting Information), including *CAMP* and *CYBA* for specific or secondary granules, *MMP9* and *ITGAM* for tertiary granules, and *CD177* and *MMP25* for secretory vesicles. These findings indicated that Clusters 1 & 4 represented subpopulations of neutrophils that were involved in degranulation. Neutrophil degranulation is characterized by releasing various granules containing enzymes and antimicrobial proteins, which play a crucial role in the body's defense against infections and inflammatory responses. Additionally, scores for phagocytosis, chemotaxis, neutrophil activation, and NADPH oxidase were elevated in Clusters 1 & 4, further confirming their functional roles (Figure S4A–D, Supporting Information).

Cluster 5 had a lower maturation score and a lower aging score, suggesting it represented the starting point of differentiation (Figure S2G, Supporting Information). Conversely, Clusters 1 & 4 displayed higher maturation scores (Figure S2G, Supporting Information) and higher expression levels of maturation‐related genes *MME* (Figure S2F, Supporting Information), indicating they were more differentiated subsets. Consistent with previous studies, in which mature neutrophils mainly utilize glycolysis for energy production,^[^ ^29^ ^]^ Clusters 1 & 4 showed higher glycolysis scores (Figure S4E, Supporting Information). Cluster 3 was characterized by higher aging scores and upregulated expression of age‐related genes, such as *CXCR4*, coupled with lower maturation scores (Figure S2F,G, Supporting Information), indicating that Cluster 3 represented an aging endpoint of differentiation.

Based on these results, we hypothesized that Cluster 5 neutrophils could shift either to aging Cluster 3 or matured, functional Clusters 1 & 4 (Figure 1F; Figure S5A, Supporting Information). The presence of MI/RI was likely to accelerate this shift, leading to an excessive abundance of activated Clusters 1 & 4, thereby exacerbating MI/RI (Figures S2D and S5B, Supporting Information). This proposed trajectory partly aligned with previous research, which identifies PMNa (degranulation group, analogous to our Clusters 1 & 4) and PMNb (ISG group, analogous to our Cluster 5) differentiating into PMNc (aging group, similar to our Cluster 3).^[^ ^28^ ^]^

We visualized the pseudotime ordering of neutrophils in Monocle split according to the neutrophil cluster (Figure 1G). Gene expression variation according to brunchtime of pseudotime was classified into 4 modules (Figure S2H, Supporting Information), with marker gene changed in these modules corresponding to the marker genes of the associated clusters (detailed in Table S6, Supporting Information).

To further confirm our conclusion, we employed the RNA velocity approach to trace cell fate and reconstruct cell lineage directions (Figure 1H; Figure S2I, Supporting Information). The latent time map generated by RNA velocity dynamics (Figure S5C, Supporting Information) was consistent with the previous pseudo‐time map. A significant transcriptional cascade was observed among genes with the highest likelihood rankings, as revealed by the dynamics of gene expression along latent time (Figure S5D, Supporting Information). High‐likelihood genes in dynamic models, such as *PROK2*, *MME*, *S100A8*, *FAM126B*, and *SLC2A3*, emerged as potential driving genes (Figure S5E, Supporting Information). In accordance with Monocle analysis, the PAGA trajectory inference map (Figure 1I) and Slingshot analysis (Figure S2J, Supporting Information) also revealed that Cluster 5 differentiates into either Cluster 3 or Clusters 1 & 4.

### 2.3 Verification of Circulating Neutrophil Clusters in Humans and Mice: Correlation of MMP9^High^ Neutrophils with MI/RI Severity

**Figure 2:**
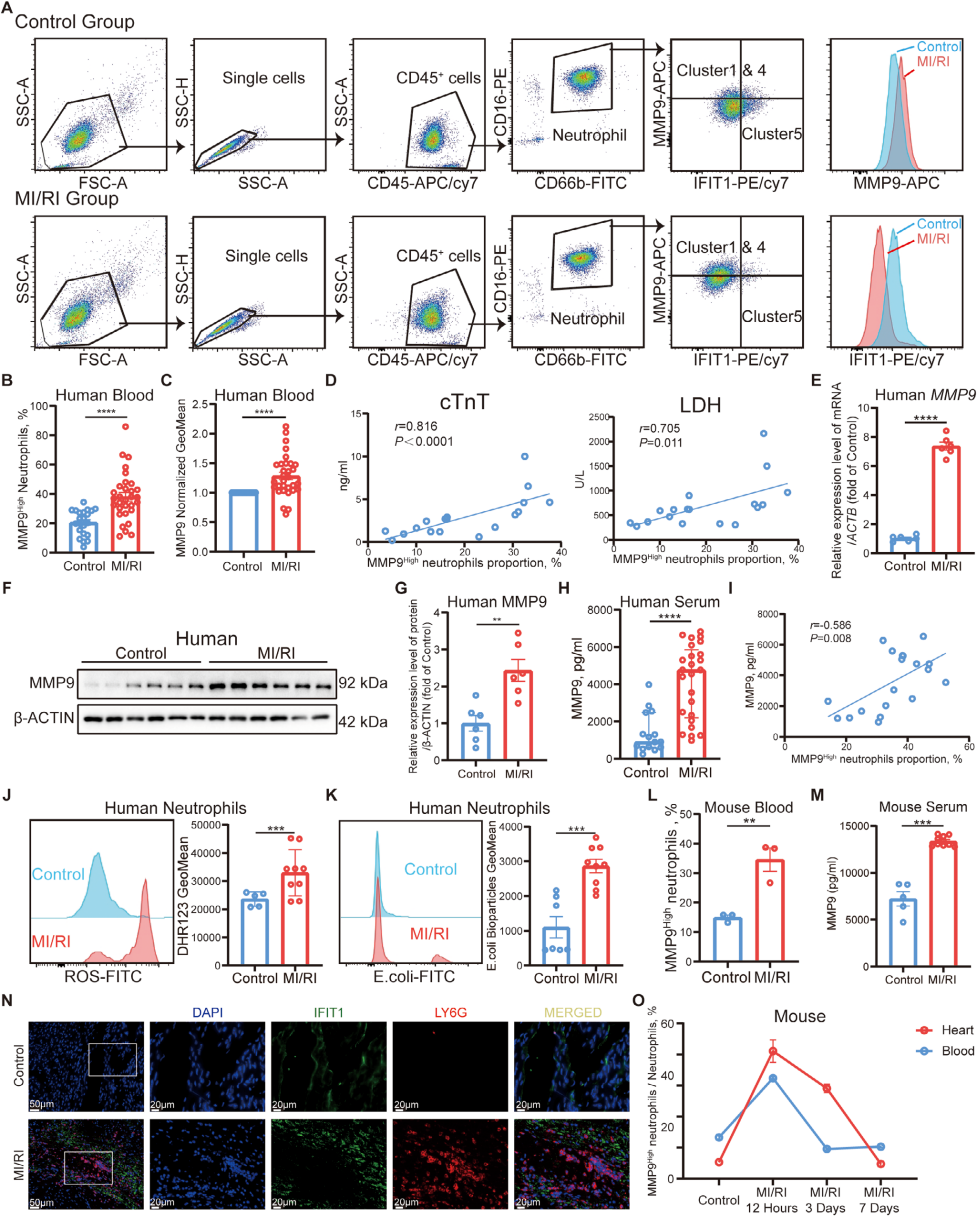
Verification of neutrophil clustering in human blood and the mouse model and the correlation of MMP9^High^ neutrophils with the severity of MI/RI. A) FACS and staining strategy for human neutrophils (CD45^+^CD66b^+^CD16^+^), Clusters 1 & 4 (MMP9^High^), and Cluster 5 (IFIT1^High^). Histogram plots to compare fluorescence density were also displayed. B) Proportion of MMP9^High^ neutrophils (unpaired *t*‐test with Welch's correction) in healthy donors (*n *= 22) and MI/RI patients (*n *= 31). C) Relative fluorescence density (one sample *t*‐test) of MMP9 in MI/RI patients (*n *= 31) compared to healthy donors (*n *= 22, normalized to 1 at same batch). D) Spearman correlation analysis of MMP9^High^ neutrophils with cTnT and LDH (*n *= 18). E) Relative mRNA levels of *MMP9* (unpaired *t*‐test with Welch's correction) of neutrophils *(n *= 6). F, G) Relative protein levels of MMP9 (unpaired *t*‐test) of neutrophils (*n *= 6). H) Concentration of MMP9 (Mann‐Whitney test) in serum of healthy donors (*n *= 15) and MI/RI patients (*n *= 25). I) Spearman correlation analysis of MMP9^High^ neutrophils with serum MMP9 (*n *= 18). J) FACs analysis of reactive oxygen species (ROS, left) production and relative fluorescence density of ROS production (right, unpaired *t*‐test with Welch's correction) in healthy donors (*n *= 9) and MI/RI patients (*n *= 5). K) FACs analysis of phagocytosis of E. coli fluorescent bioparticles (left) in neutrophil subsets and relative fluorescence density of E. coli phagocytosing (right, Mann‐Whitney test) in healthy donors (*n *= 9) and MI/RI patients (*n *= 5). L) Proportion of MMP9^High^ neutrophils (unpaired *t*‐test) in the blood of control mice (*n *= 3) and MI/RI mice (*n *= 3). M) Concentration of MMP9 (unpaired *t*‐test with Welch's correction) in serum of control mice (*n *= 5) and MI/RI mice (*n *= 10). N) Representative fluorescence images of neutrophil infiltrated in MI/RI tissue sections stained for DAPI (blue), IFIT1 (green), Ly6G (red), *200, scale bar 50 µm; *400, scale bar 20 µm. O) Proportion of MMP9^High^ neutrophils in neutrophils from blood and heart of mice along time after MI/RI (*n *= 3–6 each). All data was displayed as median with interquartile range or mean ± SEM. **p < 0.01; ***p < 0.001; ****p < 0.0001.

As functional Clusters 1 & 4 (MMP9 high‐expression neutrophils) showed unique marker genes different from MMP9 low expression neutrophils (Figure 1J), we focused on the differentiation of Cluster 5 (IFIT1 high‐expression neutrophils) to Clusters 1 & 4 (MMP9 high‐expression neutrophils). To validate the identified neutrophil subpopulations from the scRNA‐seq analysis, we collected blood from 31 patients with MI/RI and 22 healthy donors for FACS analysis (**Figure** **2**A; Figure S6A, Supporting Information). The clinical features of the subjects were detailed in Table S7 (Supporting Information). In this new cohort, we confirmed a higher proportion of MMP9^High^ neutrophils (Figure 2B) and a lower proportion of IFIT1^High^ neutrophils (Figure S6B, Supporting Information) in MI/RI patients as well as a higher relative fluorescence intensity of MMP9 (Figure 2C) and a lower relative fluorescence intensity of IFIT1 (Figure S6C, Supporting Information). After normalizing neutrophil subset proportions from MI/RI patients with health donors from the same batch, we found a positive correlation between the proportion of MMP9^High^ neutrophils and serum cardiac markers, including cardiac troponin T (cTnT) and lactate dehydrogenase (LDH, Figure 2D). The proportion of MMP9^High^ neutrophils also positively correlated with whole blood count (WBC), neutrophil count, and neutrophil‐lymphocyte ratio (NLR), and negatively correlated to platelet count (Figure S6D, Supporting Information). RNA (Figure 2E; Figure S6E, Supporting Information) and protein (Figure 2F,G) profiling data also revealed higher MMP9 levels and lower IFIT1 levels in neutrophils from MI/RI patients. MI/RI patients’ serum showed higher MMP9 concentrations (Figure 2H), which was positively associated with the proportion of MMP9^High^ neutrophils (Figure 2I), indicating that this neutrophil cluster continuously transcripted and produced MMP9 to exacerbate inflammation. Additionally, neutrophils from MI/RI patients exhibited enhanced proinflammatory functions, such as elevated production of ROS (Figure 2J) and increased phagocytosis of E. coli (Figure 2K).

In addition to human samples, we collected blood and heart samples from the MI/RI mouse model 12 h post‐surgery. Consistent with MI/RI patient data, neutrophils from MI/RI mice blood (Figure S6F, Supporting Information) exhibited a higher proportion of MMP9^High^ neutrophils (Figure 2L) and a lower proportion of IFIT1^High^ neutrophils (Figure S6G, Supporting Information). Mice that underwent MI/RI surgery also had higher plasma concentrations of MMP9 (Figure 2M). Aligning with blood data, we detected a higher proportion of MMP9^High^ neutrophils and a lower proportion of IFIT1^High^ neutrophils in heart samples from mice that underwent MI/RI surgery (Figure S6H, Supporting Information). Immunofluorescence analysis of MI/RI tissue sections corroborated our flow cytometry analysis, with Ly6G^+^ cells infiltrated into the infarcted area showing low levels of IFIT1 (Figure 2N).

To further elucidate the temporal clustering patterns of neutrophils following MI/RI, we conducted a comprehensive time‐course study. FACS analysis of neutrophils in blood and heart (Figure S6I, Supporting Information) revealed that MMP9^High^ neutrophils peaked at 12 h post‐MI/RI (Figure 2O), while IFIT1^High^ neutrophils simultaneously reached their nadir in both compartments (Figure S6J, Supporting Information). Over time, the differentiation of IFIT1^High^ neutrophils to MMP9^High^ exhibited a gradual decline. We also isolated murine neutrophils from the blood and heart by FACS sorting and extracted their mRNAs. The mRNA expression levels of *Mmp9* and *Ifit1* showed a similar trend to that observed in the flow cytometry analysis (Figure S7A,B, Supporting Information). Further time‐course study on humans also confirmed our conclusion that this neutrophil differentiation reached its peak at 12 h post‐MI/RI and gradually faded afterward (Figure S7F,G, Supporting Information).

### 2.4 Identifying MMP9^High^ Neutrophils: Key Subsets for NET Formation

**Figure 3:**
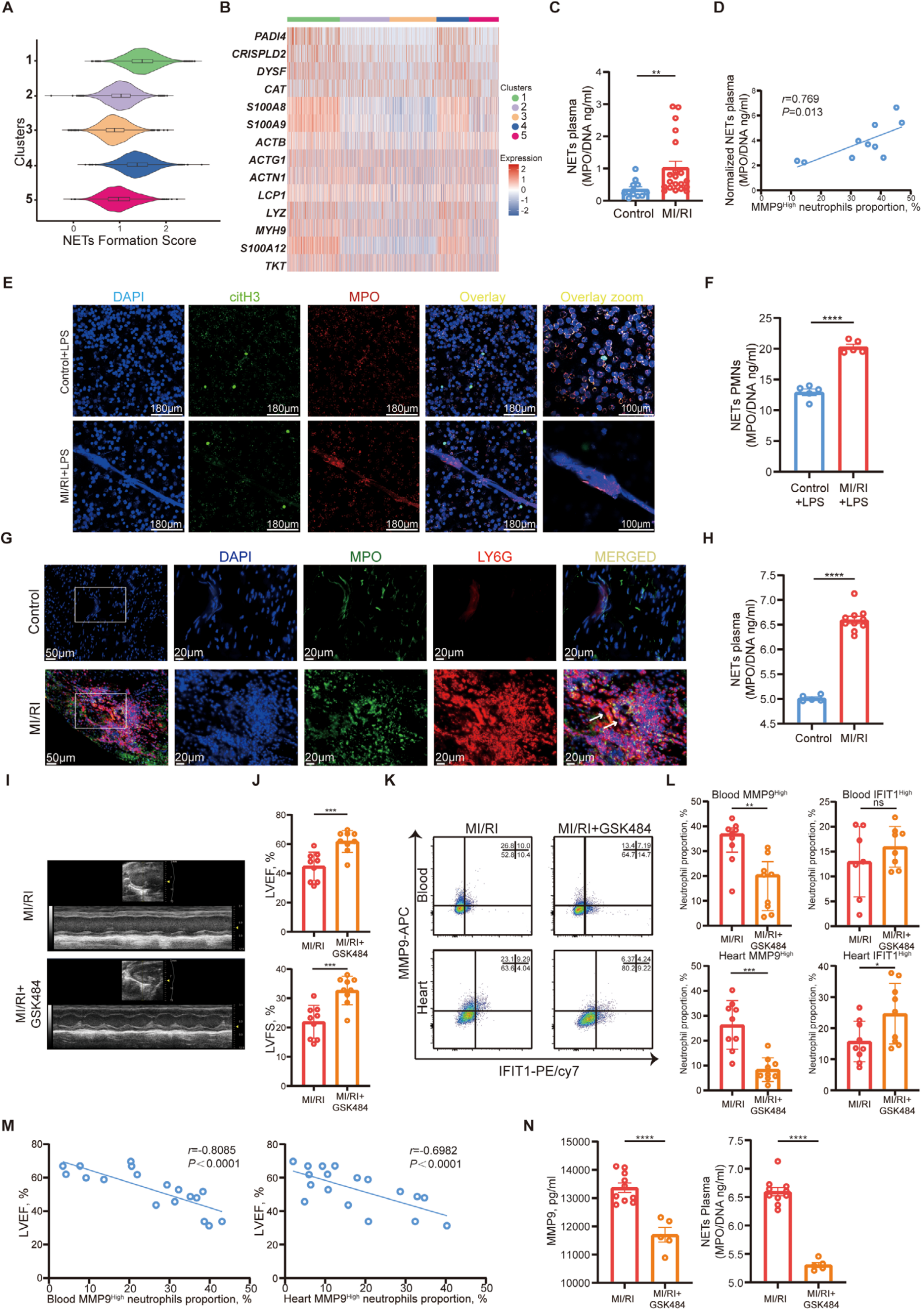
MMP9^High^ neutrophils accounted for NET formation and MI/RI. A) Violin plot of NET formation scores for each cluster. B) Heatmap of marker genes for NET formation associated with the 5 neutrophil clusters. C) The concentrations of MPO/DNA‐NETs (Mann‐Whitney test) in serum from healthy donors (*n *= 12) and MI/RI patients (*n *= 20). D) Spearman correlation analysis of MMP9^High^ neutrophils with serum MPO/DNA‐NETs (*n *= 10). E) Representative fluorescence images of NETs stained for DNA (DAPI, blue), citH3 (citH3, green), and myeloperoxidase (MPO, red) of human neutrophils from healthy donors or MI/RI patients treated with LPS, *200, scale bar 180 µm; *400, scale bar 100 µm. F) The concentrations of MPO/DNA‐NETs (unpaired *t*‐test with Welch's correction) in the neutrophil culture supernatants (*n *= 5). G) Representative fluorescence images of NET formation in MI/RI tissue sections stained for DAPI (blue), MPO (green), Ly6G (red), *200, scale bar 50 µm; *400, scale bar 20 µm. H) The concentrations of MPO/DNA‐NETs (unpaired *t*‐test) in serum from control mice (*n *= 5) and MI/RI mice (*n *= 10). I) Representative images of echocardiography of the mice undergoing MI/RI with saline or GSK484 treatment. J) LVEF (top, unpaired *t*‐test) and LVFS (bottom, unpaired *t*‐test) of MI/RI + saline mice (*n *= 9) and MI/RI + GSK484 mice (*n *= 9). K) Representative FACs images of neutrophil clustering in blood and heart of MI/RI + saline mice and MI/RI + GSK484 mice. L) Proportion of MMP9^High^ neutrophils (top left, Mann‐Whitney test) and IFIT1^High^ neutrophils (top right, unpaired *t*‐test) in the blood of MI/RI + saline mice (*n *= 9) and MI/RI + GSK484 mice (*n *= 9). The proportion of MMP9^High^ neutrophils (bottom left, unpaired *t*‐test) and IFIT1^High^ neutrophils (bottom right, unpaired *t*‐test) in the heart of MI/RI + saline mice (*n *= 9) and MI/RI + GSK484 mice (*n *= 9). M) Pearson correlation analysis of MMP9^High^ neutrophils proportion in blood with LVEF (*n *= 18) (left). Pearson correlation analysis of MMP9^High^ neutrophils proportion in heart with LVEF (*n *= 18) (right). N) Plasma concentration of MMP9 (left, unpaired *t*‐test) and MPO/DNA‐NETs (right, unpaired *t*‐test) of MI/RI + saline mice (*n *= 10) and MI/RI + GSK484 mice (*n *= 5). All data was displayed as median with interquartile range or mean ± SEM. NS, not significant, *p* > 0.05; *p < 0.05; **p < 0.01; ***p < 0.001; ****p < 0.0001.

Given that *PADI4*, a hallmark gene for NET formation,^[^ ^30^ ^]^ is highly expressed in MMP9^High^ neutrophils (Clusters 1 & 4), we hypothesized that MMP9^High^ neutrophils constitute a specific subset involved in NET formation. Using AddModuleScore (Scoring Marker detailed in Table S5, Supporting Information), we observed higher NET formation scores (**Figure** **3**A; Figure S8A, Supporting Information) and elevated levels of genes related to NET formation in Cluster 1 & 4 (Figure 3B; Figure S8B, Supporting Information).

Comparing NET formation between neutrophils from MI/RI patients and healthy donors, we found higher plasma levels of MPO/DNA‐NETs in MI/RI patients (Figure 3C), positively correlating with the proportion of MMP9^High^ neutrophils (Figure 3D). In vitro experiments showed that, without any treatment, neutrophils from MI/RI patients and healthy donors exhibited minimal NET formation (Figure S8C–F, Supporting Information). However, neutrophils from patients spontaneously formed small amounts of NETs, which were colocalized with MMP9 (Figure S8G, Supporting Information). PMA stimulation induced substantial NET formation in both groups (Figure S8C–F), while LPS stimulation resulted in more NETs in neutrophils from patients compared to healthy donors (Figure 3E,F; Figure S8E,F, Supporting Information). Immunofluorescence of MI/RI tissue from mice showed NET formation in neutrophils within the heart (Figure 3G) and higher plasma MPO/DNA‐NET levels in MI/RI mice (Figure 3H).

**Figure 4:**
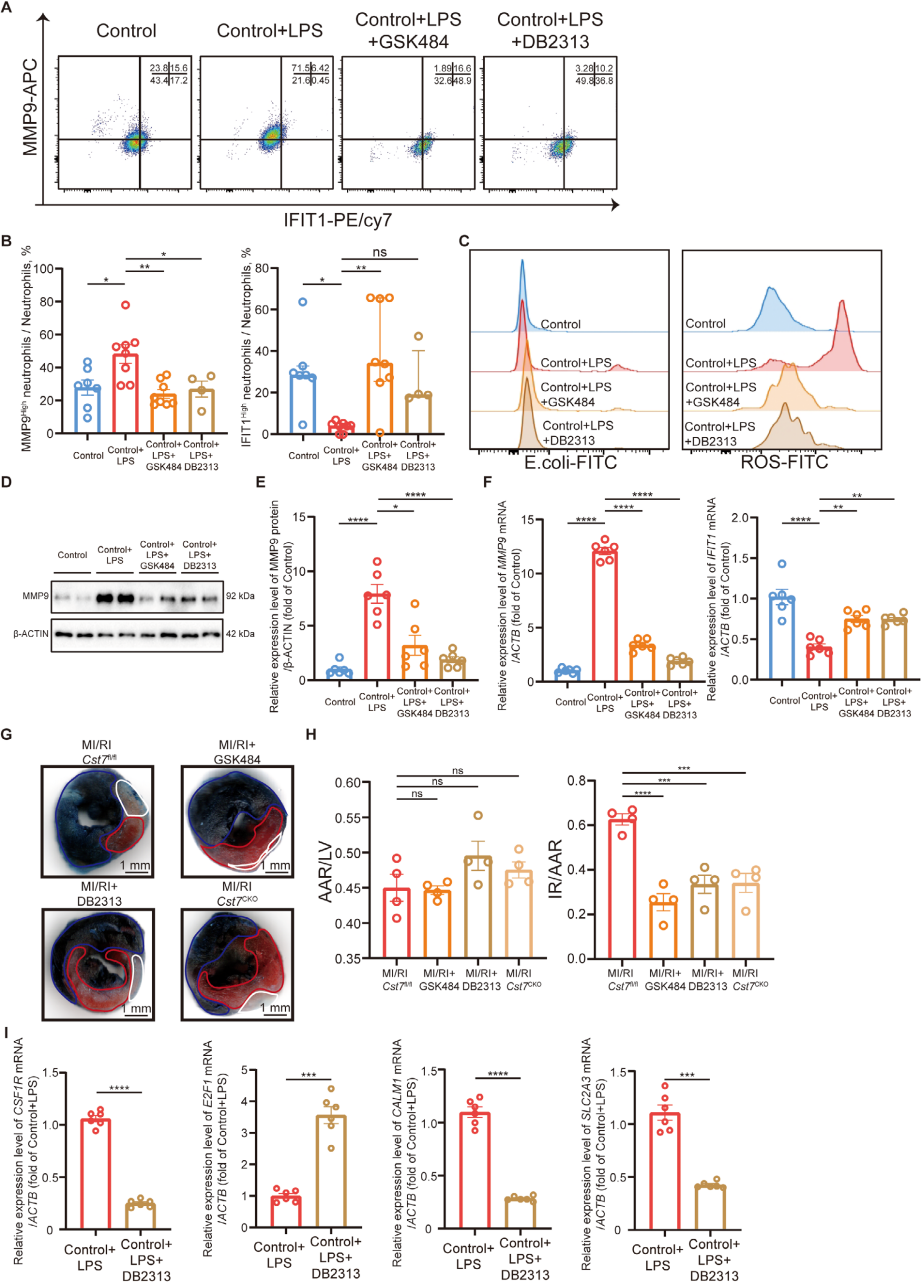
GSK484, DB2313, or *CST7* ^CKO^ can decrease the proportion of MMP9^High^ neutrophils and weaken their function in vitro and in vivo. A) Representative FACs images of neutrophil clustering of neutrophils from the blood of healthy donors. B) Proportion of MMP9^High^ neutrophils (left, one‐way ANOVA test with Tukey's multiple comparisons test) and IFIT1^High^ neutrophils (right, Kruskal‐Wallis's test with Dunn's multiple comparisons test) in neutrophil from the blood of healthy donors (control + vehicle) (*n *= 7), control + LPS+ vehicle (*n *= 8), control + LPS + GSK484 (*n *= 8), and control + LPS + DB2313 (*n *= 4). C) FACs analysis of phagocytosis of E. coli fluorescent bioparticles in neutrophil subsets and reactive oxygen species production. D, E) Relative protein levels of MMP9 (one‐way ANOVA test with Tukey's multiple comparisons test) of neutrophils (*n *= 4). F) Relative mRNA levels of *MMP9* (left, one‐way ANOVA test with Tukey's multiple comparisons test) and *IFIT1* (right, one‐way ANOVA test with Tukey's multiple comparisons test) of neutrophils (*n *= 6). G) Representative image of left ventricular tissue sections stained with Evans blue and 2,3,5‐triphenyl tetrazolium chloride at 12 h after MI/RI to delineate the area at risk (AAR, red) and the infarcted area (IR, white) (scale bar, 1 mm). H) The ratios of AAR/LV (left, one‐way ANOVA test with Tukey's multiple comparisons test) and IR/AAR (right, one‐way ANOVA test with Tukey's multiple comparisons test) were compared (*n *= 4). I) Relative mRNA levels of *CSF1R* (top left, unpaired *t*‐test), *E2F1* (top right, unpaired *t*‐test with Welch's correction), *CALM1* (bottom left, unpaired *t*‐test with Welch's correction), and *SLC2A3* (bottom right, unpaired *t* test with Welch's correction) of neutrophils (*n *= 6). All data was displayed as median with interquartile range or mean ± SEM. NS, not significant, *p* > 0.05; *p < 0.05; **p < 0.01; ***p < 0.001; ****p < 0.0001.

As PADI4 plays a pivotal role in mediating NET formation, we explored the effects of the selective PADI4 inhibitor GSK484, known to efficiently block NET formation and treat NET‐related diseases.^[^ ^31^, ^32^ ^]^ GSK484 inhibited LPS‐induced NET formation in neutrophils from MI/RI patients (Figure S9A–D, Supporting Information), reduced MMP9^High^ neutrophils, increased IFIT1^High^ neutrophils, and lowered proinflammatory function (**Figure** **4**A–C). Western and PCR data (Figure 4D–F) confirmed the reduced MMP9 expression and elevated IFIT1 expression, while immunofluorescence demonstrated reduced MMP9^High^ neutrophils and colocalized NET formation with GSK484 treatment (Figure S9E, Supporting Information). In vivo, GSK484 administration improved MI/RI outcomes in mice, evidenced by improved left ventricular ejection fraction (LVEF), improved left ventricular fractional shortening (LVFS) (Figure 3I,J), and reduced infarcted size (Figure 4G,H). FACS analysis showed decreased MMP9^High^ neutrophils and increased IFIT1^High^ neutrophils in GSK484‐treated mice (Figure 3K,L). The proportion of MMP9^High^ neutrophils negatively correlated with LVEF (Figure 3M). GSK484‐treated mice had lower serum MMP9 and MPO/DNA‐NETs levels (Figure 3N). Immunofluorescence revealed reduced NETs and increased IFIT1 in neutrophils after GSK484 treatment (Figure S10A,B, Supporting Information). GSK484 can also reduce adverse tissue remodeling 7 days after MI/RI (Figure S11, Supporting Information).

These data indicated that MMP9^High^ neutrophils accounted for NET formation, and NET formation inhibitors can ameliorate MI/RI injury by reducing the proportion of MMP9^High^ neutrophils. Thus, we elucidated a fundamental mechanism underlying MI/RI and, for the first time, identified the role of MMP9^High^ neutrophils in orchestrating the formation of harmful NETs and deteriorating MI/RI.

### 2.5 Transcription Factor SPI1 was Essential in the Differentiation of Neutrophil Clusters and NET Formation

**Figure 5:**
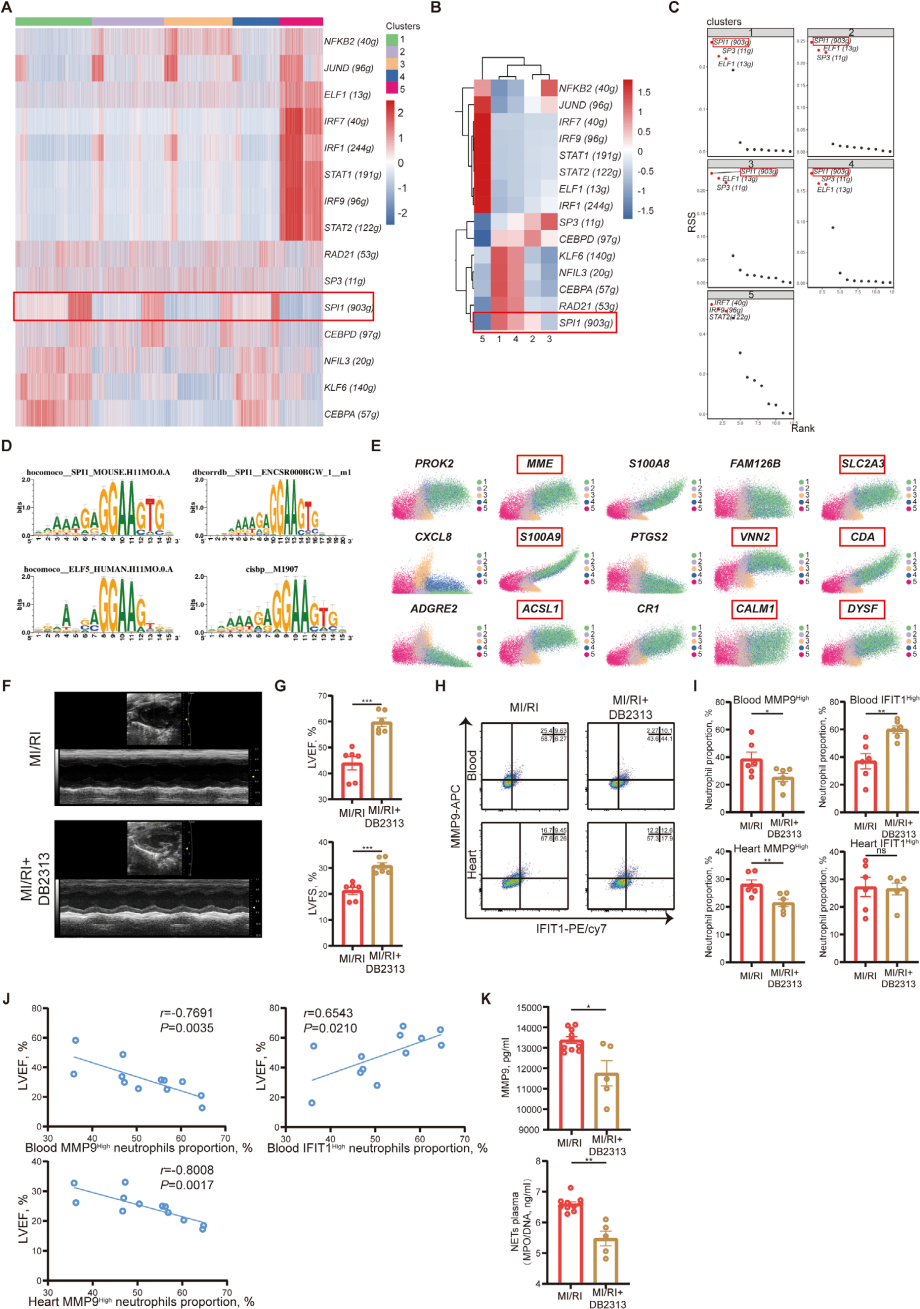
Transcription factor SPI1 was essential in the differentiation of neutrophil clusters and NET formation. A) Heatmap of regulon activity according to 5 clusters. B) Heatmap of regulon activity. C) Ranking plot of regulon specificity score (RSS) for each cluster. D) High confidence binding motif of transcription factor SPI1. E) Top 15 likelihood driving genes alone time grouped by clusters. The red boxes mark the genes which might be regulated by SPI1. F) Representative images of echocardiography of the mice undergoing MI/RI with saline or DB2313 treatment. G) LVEF (top, unpaired *t*‐test) and LVFS (bottom, unpaired *t*‐test) of MI/RI + saline mice (*n *= 6) and MI/RI + DB2313 mice (*n *= 6). H) Representative FACs images of neutrophil clustering in blood and heart of MI/RI + saline mice and MI/RI + DB2313 mice. I) Proportion of MMP9^High^ neutrophils (top left, unpaired *t*‐test) and IFIT1^High^ neutrophils (top right, unpaired *t*‐test) in the blood of MI/RI + saline mice (*n *= 6) and MI/RI + DB2313 mice (*n *= 6). Proportion of MMP9^High^ neutrophils (bottom left, unpaired *t*‐test) and IFIT1^High^ neutrophils (bottom right, unpaired *t*‐test) in heart of MI/RI + saline mice (*n *= 6) and MI/RI + DB2313 mice (*n *= 6). J) Pearson correlation analysis of MMP9^High^ neutrophils proportion in blood with LVEF (*n *= 12) (top left). Pearson correlation analysis of IFIT1^High^ neutrophils proportion in blood with LVEF (*n *= 12) (top right). Pearson correlation analysis of MMP9^High^ neutrophils proportion in heart with LVEF (*n *= 12) (bottom). K) Plasma concentration of MMP9 (left, unpaired *t*‐test with Welch's correction) and MPO/DNA‐NETs (right, unpaired *t*‐test with Welch's correction) of MI/RI + saline mice (*n *= 10) and MI/RI + DB2313 mice (*n *= 5). All data was displayed as mean ± SEM. NS, not significant, *p* > 0.05; *p < 0.05; **p < 0.01; ***p < 0.001.

To further explore the mechanism of neutrophil differentiation, particularly the shift from IFIT1^High^ to MMP9^High^ neutrophils, we performed single‐cell regulatory network inference and clustering (SCENIC) analysis. We found that transcription factor SPI1 had higher regulon activity and specificity scores in Clusters 1 & 4 (**Figure** **5**A–C), suggesting its critical role in neutrophil differentiation.

SPI1, also known as PU.1, is vital for neutrophil maturation.^[^ ^33^ ^]^ It binds to DNA at consensus sites with a 5′‐GGAA/T‐3′ motif (Figure 5D). RNA velocity analysis showed that several driving genes containing this motif, such as *CALM1* and *SLC2A3*, might be regulated by SPI1 (Figure 5E, detailed in Table S8, Supporting Information). The expression levels of *Spi1* and *Calm1* mRNA in neutrophils from blood and heart in mice post‐MI/RI followed the same trend as *Mmp9*, peaking at 12 h post‐MI/RI (Figure S7C,D, Supporting Information).

Small molecule DB2313 inhibits SPI1 binding by interacting with the minor groove of AT‐rich tracks.^[^ ^34^, ^35^ ^]^ In vitro data showed it inhibited *CSF1R* transcription while promoting *E2F* (Figure 4I).^[^ ^34^ ^]^ DB2313 also inhibited *CALM1* and *SLC2A3* transcription (Figure 4I) and prevented the differentiation of IFIT1^High^ neutrophils into MMP9^High^ neutrophils, reducing their pro‐inflammatory response to LPS (Figure 4A–C). Additionally, DB2313 reduced NET formation and the expression level of PADI4 (Figure S9A–D), inhibited the elevation of MMP9, and reduction of IFIT1 after LPS stimulation (Figure 4D‐F; Figure S9E, Supporting Information).

In a murine MI/RI model, DB2313 ameliorated the decrease in LVEF and LVFS (Figure 5F,G) and reduced the infarct area (Figure 4G,H). FACS analysis showed fewer MMP9^High^ neutrophils and more IFIT1^High^ neutrophils (Figure 5H,I) in mice treated with DB2313. The proportion of MMP9^High^ neutrophils negatively correlated with LVEF, while the proportion of IFIT1^High^ neutrophils positively correlated with LVEF (Figure 5J). DB2313 also decreased serum levels of MMP9 and MPO/DNA‐NETs (Figure 5K). Immunofluorescence analysis of MI/RI tissues showed reduced NET formation and higher IFIT1 levels in neutrophils post‐DB2313 treatment (Figure S10A,B, Supporting Information). DB2313 can also reduce adverse tissue remodeling 7 days after MI/RI (Figure S11, Supporting Information).

These data demonstrated that inhibiting transcription factor SPI1 by DB2313 hindered the differentiation of IFIT1^High^ neutrophils into MMP9^High^ neutrophils, which in turn mitigated NET‐mediated damage, offering a potential therapeutic strategy for MI/RI injury.

### 2.6 The SPI1/CST7 Axis in MMP9^High^ Neutrophils and NET Formation: A Therapeutic Target for MI/RI

**Figure 6:**
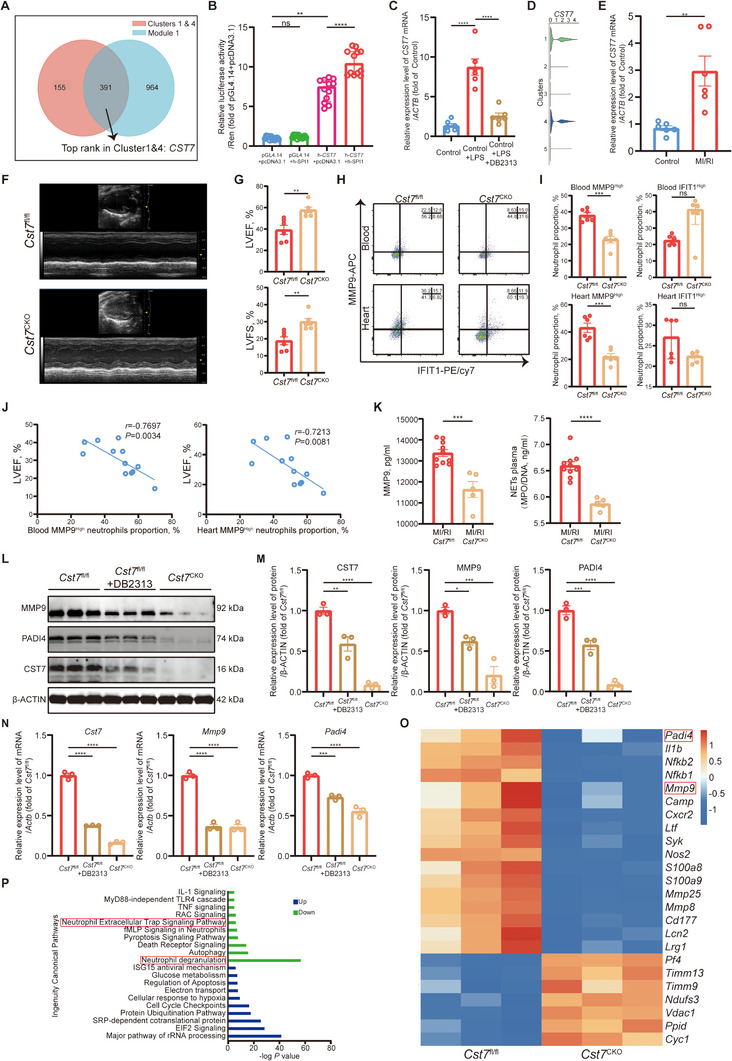
*CST7* was a key marker gene in MMP9^High^ neutrophils regulated by SPI1. A) Veen diagram of marker genes in Cluster 1 & 4 and genes regulated by TF CSI module 1. B) SPI1 and *CST7* promoter luciferase activity were detected by dual‐luciferase assays (*n *= 12 each, Kruskal‐Wallis's test with Dunn's multiple comparisons test). C) Relative mRNA levels of *CST7* of human neutrophils underwent vehicle control (*n *= 6), vehicle control + LPS (*n *= 6), or DB2313 + LPS (*n *= 6) treatment (one‐way ANOVA test with Tukey's multiple comparisons test). D) Violin plot of *CST7* expression level of each cluster. E) Relative mRNA levels of *CST7* (unpaired *t*‐test) of human neutrophils (*n *= 6). F) Representative images of echocardiography from the *Cst7* ^fl/fl^ and *Cst7* ^CKO^ mice undergoing MI/RI. G) LVEF (top, unpaired *t*‐test) and LVFS (bottom, unpaired *t*‐test) of *Cst7* ^fl/fl^ + MI/RI mice (*n *= 6) and *Cst7* ^CKO^ + MI/RI mice (*n *= 6). H) Representative FACs images of neutrophil clustering in blood and heart of *Cst7* ^fl/fl^ + MI/RI mice and *Cst7* ^CKO^ + MI/RI mice. I) Proportion of MMP9^High^ neutrophils (top left, unpaired *t*‐test) and IFIT1^High^ neutrophils (top right, Mann‐Whitney test) in the blood of *Cst7* ^fl/fl^ + MI/RI mice (*n *= 6) and *Cst7* ^CKO^ + MI/RI mice (*n *= 6). Proportions of MMP9^High^ neutrophils (bottom left, unpaired *t*‐test) and IFIT1^High^ neutrophils (bottom right, Mann‐Whitney test) in hearts of *Cst7* ^fl/fl^ + MI/RI mice (*n *= 6) and *Cst7* ^CKO^ + MI/RI mice (*n *= 6). J) Pearson correlation analysis of the proportion of blood MMP9^High^ neutrophils with LVEF (*n *= 12) (left). Pearson correlation analysis of the proportion of heart MMP9^High^ neutrophils with LVEF (*n *= 12) (right). K) Plasma concentration of MMP9 (left, unpaired *t*‐test) and MPO/DNA‐NETs (right, unpaired *t*‐test) in *Cst7* ^fl/fl^ + MI/RI mice (*n *= 10) and *Cst7* ^CKO^ + MI/RI mice (*n *= 5). L, M) Relative protein levels of CST7 (left), MMP9 (middle), and PADI4 (right) of neutrophils (*n *= 3, one‐way ANOVA test with Tukey's multiple comparisons test for all). N) Relative mRNA levels of *Cst7* (left), *Mmp9* (middle), and *Padi4* (right) of neutrophils (*n *= 3, one‐way ANOVA test with Tukey's multiple comparisons test for all). O) Heatmap of the concerned 25 DEGs from the RNA‐seq result of neutrophils from *Cst7* ^CKO^ mice versus *Cst7* ^fl/fl^ mice. P) IPA analysis of DEGs from the RNA‐seq result of neutrophils from *Cst7* ^CKO^ mice versus *Cst7* ^fl/fl^ mice. All data was displayed as median with interquartile range or mean ± SEM. NS, not significant, *p *> 0.05; *p < 0.05; **p < 0.01; ***p < 0.001; ****p < 0.0001.

To identify key downstream genes regulated by SPI1, we employed the connection specificity index (CSI) of regulons. We found that SPI1 may regulate downstream genes together with CEBPD, KLF6, CEBPA, and NFIL3, which combined as transcription module 1, with high transcriptional activity in Clusters 1 & 4 (Figure S12A,B, Supporting Information). Through a combined analysis of marker genes in Clusters 1 & 4 and their potential transcriptional regulation by transcription factors in module 1, we identified *CST7* as a key marker gene in Clusters 1 & 4 (**Figure** **6**A, detailed in Table S9, Supporting Information). *CST7* encodes Cystatin F, which is upregulated in neutrophils during acute inflammation.^[^ ^36^ ^]^ Dual‐luciferase assays and ChIP‐qPCR assays confirmed SPI1's regulation of *CST7* (Figure 6B; Figure S12C, Supporting Information). DB2313 administration inhibited LPS‐induced *CST7* transcription in human circulating neutrophils (Figure 6C). *CST7* expression in Clusters 1 & 4 was confirmed via violin and UMAP plots (Figure 6D; Figure S12D, Supporting Information). RNA extracted from the patient's neutrophils also showed elevated *CST7* levels (Figure 6E). In mice, *Cst7* mRNA level in neutrophils from blood and heart post‐MI/RI correlated with *Spi1* levels, peaking at 12 h post‐injury (Figure S7E, Supporting Information).

To further confirm CST7's role in MI/RI, we generated neutrophil‐specific *Cst7* knockout mice (*Cst7* ^CKO^), using *Cst7* ^fl/fl^ mice as controls. *Cst7* knockout improved LVEF and LVFS (Figure 6F,G) and reduced the infarct area (Figure 4G,H) in MI/RI mice. FACS analysis showed fewer MMP9^High^ neutrophils in *Cst7* ^CKO^ mice (Figure 6H,I). The proportion of MMP9^High^ neutrophils negatively correlated with LVEF (Figure 6J). Immunofluorescence indicated reduced NET formation and higher IFIT1 levels in *Cst7* ^CKO^ neutrophils (Figure S10A,B, Supporting Information). Additionally, serum levels of MMP9 and MPO/DNA‐NETs were lower in *Cst7* ^CKO^ mice post‐MI/RI (Figure 6K). *Cst7* ^CKO^ can also reduce adverse tissue remodeling 7 days after MI/RI (Figure S11, Supporting Information). Treatment with DB2313 did not further improve MI/RI outcomes or reduce MMP9^High^ neutrophils in *Cst7* ^CKO^ mice (Figure S12E–H, Supporting Information), suggesting that SPI1 regulates the MMP9^High^ neutrophil subpopulation predominantly through CST7.

To obtain mature neutrophils as much as possible, we chose peritoneal neutrophils for in vitro experiments. Peritoneal neutrophils from *Cst7* ^CKO^ mice showed decreased PADI4 and MMP9 in both mRNA and protein levels (Figure 6L–N). Upon analysis of the DEGs from the RNA‐seq result, we found that neutrophil degranulation and NET formation pathways and related genes *Padi4* and *Mmp9* were significantly downregulated in the *Cst7* ^CKO^ peritoneal neutrophils compared to those in the *Cst7* ^fl/fl^ cells (Figure 6O,P). Therefore, CST7 played a vital role in neutrophil degranulation and NET formation.

**Figure 7:**
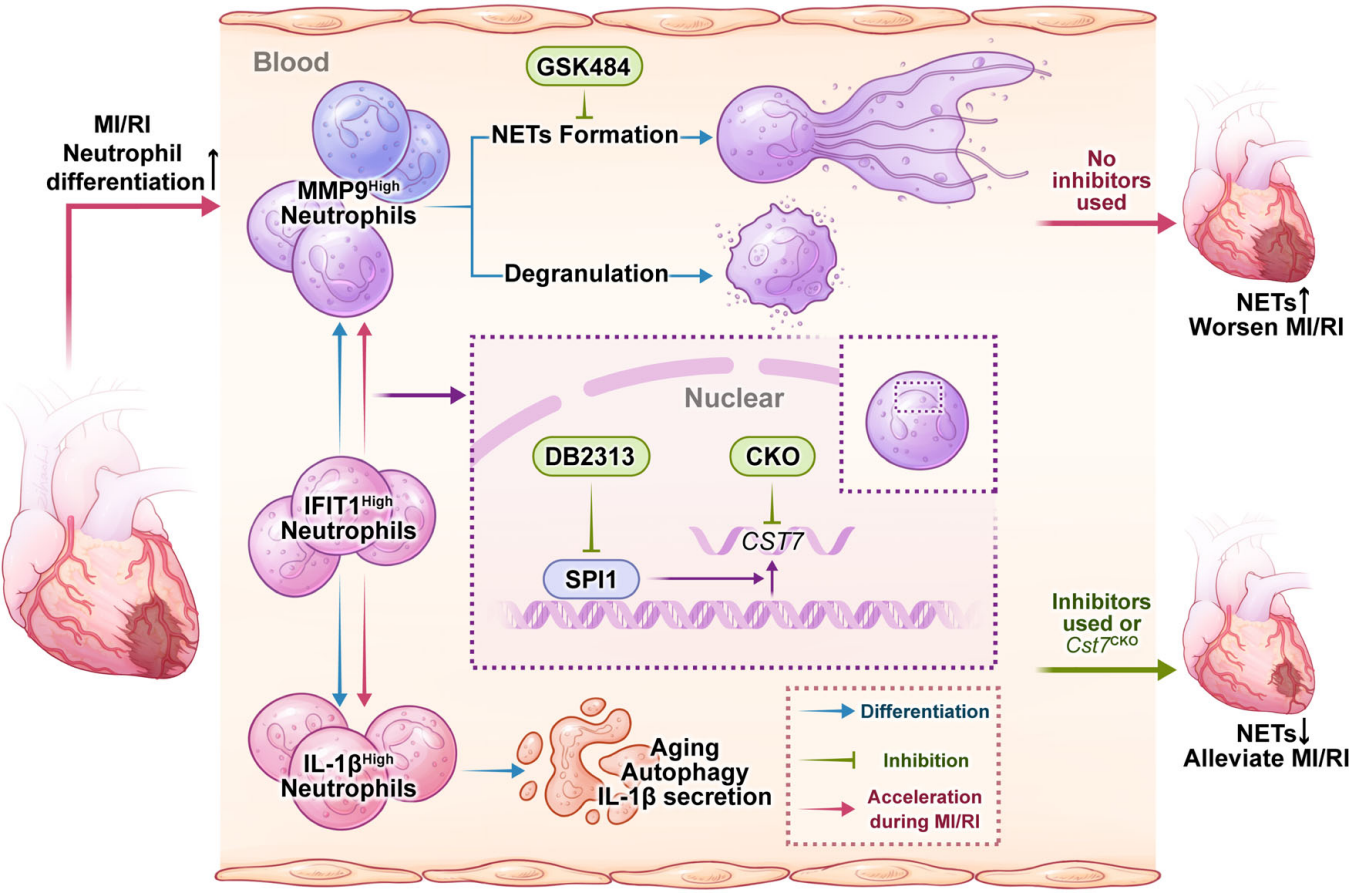
Differentiation of IFIT1^High^ neutrophils to MMP9^High^ neutrophils accelerate during MI/RI by SPI1/CST7 pathway and exacerbate MI/RI by NET formation and degranulation. Cluster 5 (IFIT1^High^) neutrophils differentiate into Cluster 1 & 4 (MMP9^High^) neutrophils and Cluster 3 (IL‐1β^High^) neutrophils, respectively. MMP9^High^ neutrophils mainly account for NET formation. During MI/RI, this differentiation process is accelerated and MMP9^High^ neutrophils can exacerbate MI/RI by forming NETs. The differentiation process of IFIT1^High^ neutrophils to MMP9^High^ neutrophils is mainly regulated by the transcription factor SPI1 and its downstream marker gene *CST7*. Administration of GSK484 (PADI4 inhibitor), DB2313 (SPI1 inhibitor), or depletion of *CST7* can reduce MMP9^High^ neutrophils and NET formation, thus ameliorating MI/RI. NETs, neutrophil extracellular traps; MI/RI, myocardial ischemia/reperfusion injury.

In conclusion, we have identified an important SPI1/CST7 regulatory pathway in neutrophil differentiation. Targeting this pathway can significantly reduce MMP9^High^ neutrophil subpopulations and NET formation, thereby ameliorating MI/RI (**Figure** **7**).

## 3 Discussion

Neutrophils play a pivotal role in mediating innate immune responses, and the release of NETs is involved in various pathological conditions, including ischemia‐reperfusion injury, infection, autoimmune diseases, and tumor metastasis.^[^ ^7^, ^8^ ^]^ Despite extensive research identifying neutrophil heterogeneity and key cellular pathways involved in NET formation under these diverse conditions, the critical relationship between specific neutrophil subpopulations and NET formation remains elusive. Additionally, as the majority of neutrophil research remains focused on mice, studies on human neutrophil subpopulations and their functions are relatively underexplored.

Our primary discovery is the identification of two neutrophil precursor subpopulations in humans, termed MMP9 high‐expression neutrophils (Clusters 1 & 4), which significantly contribute to NET formation. These MMP9^High^ neutrophils exhibit an enhanced capacity for both NET formation and tissue damage in MI/RI. MMP9, a zinc‐dependent endopeptidase, is critical in extracellular matrix remodeling and is implicated in various cardiovascular pathologies, including atherosclerosis, aneurysm formation, and post‐infarction remodeling. Additionally, as a component of tertiary granules in neutrophils, MMP9 has also been identified as having a distinct role in enhancing NET formation,^[^ ^37^ ^]^ tissue damage,^[^ ^38^ ^]^ and NET‐mediated liver reperfusion injury.^[^ ^39^ ^]^ However, growing evidence also highlights selected MMPs' unexpected physiological and pathological roles in intracellular compartments,^[^ ^40^, ^41^ ^]^ apart from their function as granular components. Our results further analyze the unique functions of intracellular MMP9, and underscore the importance of MMP9 as a factor driving the functional heterogeneity of neutrophils, suggesting its pivotal role in exacerbating and potentially mediating immune responses during ischemic events.

Specifically, we define five distinct clusters of neutrophils in the blood of MI/RI patients and explored their functions and differentiation pathways. Type I interferon response Cluster 5 (IFIT1^High^ neutrophils) can differentiate either to mature and proinflammatory Clusters 1 & 4 (MMP9^High^ neutrophils) or aging Cluster 3. Cluster 2 may represent an intermediate state of differentiation. These findings provide a comprehensive understanding of neutrophil subpopulations and their roles in MI/RI, offering potential targets for therapeutic intervention.

Additional experiments from 31 MI/RI patients and 22 healthy donors and MI/RI mice confirme our findings that MI/RI can increase the proportion of MMP9^High^ neutrophils and decrease the proportion of IFIT1^High^ neutrophils. This finding was further validated in blood and heart samples from MI/RI mice. MMP9^High^ neutrophils could serve as potent markers to reflect the severity of MI/RI and neutrophil mobilization and activation.

In a time course study, we found that MMP9^High^ neutrophils mainly peak at 12 h to 1 day in both human and mouse blood and heart. However, no significant difference was found in the mouse bone, possibly because emergency granulopoiesis in the bone marrow mainly manifests in the differentiation and proliferation of progenitor cells.^[^ ^42^ ^]^ Mature neutrophils are released to the periphery blood in a unified subpopulation ratio,^[^ ^43^ ^]^ where further subpopulation differentiation occurs as described in our study.

For the first time, we have identified MMP9^High^ neutrophils as a critical subset that regulates the formation of NETs, which is essential for promoting MI/RI. In severe infection models like sepsis,^[^ ^20^ ^]^ most neutrophils, due to excessive stimulation, have already undergone NETosis, making it challenging to capture neutrophils at a stage capable of forming NETs. However, MI/RI, a sterile inflammation with weaker stimulation, fortunately, provides an opportunity to capture a subset of neutrophils that are undergoing NET formation. This provides a critical direction for studying neutrophil NETs not only in MI/RI but also in other diseases related to NETs and innate immunity, such as sepsis, pneumonia, and cancer.

The neutrotime transcriptional signature defines a continuous spectrum of neutrophils across various biological compartments.^[^ ^44^ ^]^ Thus, to further elucidate the mechanisms regulating the differentiation of neutrophil subsets, we conducted SCENIC analysis and identified an important transcription factor, SPI1. SPI1 is reported to regulate the processes of neutrophil differentiation and maturation.^[^ ^45^, ^46^ ^]^ Elevated SPI1 expression level in inflammatory neutrophils is detected in both human and murine models, especially in the presence of inflammatory stimuli.^[^ ^47^, ^48^ ^]^ Our study prolonged the known function of SPI1 to mature circulating neutrophils, identifying it as a key transcription factor that facilitates neutrophil differentiation to MMP9 high expression state through a key marker gene CST7. This SPI1/CST7 signaling pathway is critical for neutrophil differentiation and NET formation.

Unlike previous studies on neutrophil‐macrophage intercellular communication in cardiovascular disease,^[^ ^49^, ^50^ ^]^ we did not find that our inhibition of MMP9^High^ neutrophils and NET formation affected macrophage infiltration and polarization at 12 h and 3 days after MI/RI. These researches suggest that neutrophils mainly help macrophages maintain their efferocytotic phenotype during the repair period of 5–7 days after MI/RI to engulf apoptotic cardiomyocytes^[^ ^49^ ^]^ or in chronic inflammation, such as atherosclerosis.^[^ ^50^ ^]^ Our study suggests that reducing NETs‐related inflammation by inhibiting neutrophil subpopulation differentiation in the early stages of MI/RI can improve MI/RI independently of early macrophage polarization changes. In the future, more comprehensive research on the overall immune microenvironment targeting neutrophil‐macrophage intercellular communication is worth exploring.

In conclusion, our findings reveal that SPI1, by regulating CST7, mediates the transition of IFIT1^High^ neutrophils to MMP9^High^ neutrophils, elucidating the molecular mechanisms underlying neutrophil subsets transition and NET formation. This not only provides new insight into the pathological mechanisms of MI/RI but also offers a theoretical basis for developing therapeutic strategies targeting the SPI1/CST7 pathway. In addition, MMP9^High^ neutrophils, which are crucial for NET formation, can be a novel intervention target.

## 4 Experimental Section

### Patients

Informed consent was received as per the Declaration of Helsinki. To eliminate the influence of diurnal oscillation on neutrophils and cater to the majority of acute myocardial syndrome (ACS) onset times, ACS was uniformly selected that occurred between 18–24 o'clock, with an onset time less than 12 h before PCI surgery, and collected blood samples 12 h after onset, simultaneously collect blood samples from the control group. The first 3 samples of the MI/RI group and 3 samples of the control group were sent for scRNA‐seq, and the other samples were used for verification study. For the time‐course study, 5 blood samples were collected at each time point: 12 h, 24 h, and 3 days after MI/RI. The study was approved by the Ethics Committee of the Fudan University Zhongshan Hospital, China (approval number: B2023‐143R), with informed consent obtained from all donors.

### Human Sample Collection

The blood was collected in an EDTA anticoagulant tube and transported at 4 °C to inhibit the activation of neutrophils. Neutrophils were isolated from blood using EasySep Direct Human Neutrophil Isolation Kit (19 666, Stemcell) and prepared for the flowing experiments. The rest blood was kept at room temperature for 20–30 min and then centrifugated at 1500 g for 20 min to collect the serum.

### Single‐Cell Collection, Library Construction, and Sequencing

Isolated human neutrophils were sorted into PBS containing 0.05% BSA following the 10× Genomics protocol. The cell preparation time before loading onto the 10× Chromium controller was <2 h. Cell viability and counting were evaluated with trypan blue by microscopy, and samples with viabilities >85% were used for sequencing. Libraries were constructed using the Single Cell 3′ Library Kit V2 (10× Genomics). Transcriptome profiles of individual cells were determined by 10× Genomics‐based droplet sequencing. Once prepared, indexed complementary DNA (cDNA) libraries were sequenced with paired‐end reads on an Illumina NovaSeq 6000 (Illumina).

### Single‐Cell RNA‐Seq Data Preprocessing

The Cell Ranger software pipeline (version 5.0.0) provided by 10×Genomics was used to demultiplex cellular barcodes, map reads to the genome and transcriptome using the STAR aligner, and down‐sample reads as required to generate normalized aggregate data across samples, producing a matrix of gene counts versus cells. The unique molecular identifier (UMI) was processed count matrix using the R package Seurat^[^ ^51^ ^]^ (version 4.0.0). To remove low‐quality cells and likely multiplet captures, which is a major concern in microdroplet‐based experiments, a criterion was applied to filter out cells with UMI/gene numbers out of the limit of median value ± 2‐fold of median absolute deviations assuming a Gaussian distribution of each cells' UMI/gene numbers. Following a visual inspection of the distribution of cells by the fraction of mitochondrial genes expressed, low‐quality cells were further discarded where >10% of the counts belonged to mitochondrial genes. Additionally, the DoubletFinder package^[^ ^52^ ^]^ (version 2.3.0) was applied to identify potential doublets. After applying these QC criteria, 32 291 single cells were included in downstream analyses. Library size normalization was performed with the NormalizeData function in Seurat^[^ ^51^ ^]^ to obtain the normalized count. Specifically, the global‐scaling normalization method “LogNormalize” normalized the gene expression measurements for each cell by the total expression, multiplied by a scaling factor (10000 by default), and the results were log‐transformed.

Top variable genes across single cells were identified using the method described before.^[^ ^53^ ^]^ The most variable genes were selected using the FindVariableGenes function (mean. function = FastExpMean, dispersion. function = FastLogVMR) in Seurat.^[^ ^51^ ^]^ To remove the batch effects in single‐cell RNA sequencing data, the mutual nearest neighbors (MNN) presented by Haghverdi et al.^[^ ^54^ ^]^ was performed with the R package bachelor. Graph‐based clustering was performed to cluster cells according to their gene expression profile using the FindClusters function in Seurat.^[^ ^51^ ^]^ Cells were visualized using a 2‐D Uniform Manifold Approximation and Projection (UMAP) algorithm with the RunUMAP function in Seurat.^[^ ^51^ ^]^ The FindAllMarkers function was used (test. use = bimod) in Seurat^[^ ^51^ ^]^ to identify marker genes of each cluster. For a given cluster, FindAllMarkers identified positive markers compared with all other cells. Then, the R package SingleR,^[^ ^55^ ^]^ a novel computational method for unbiased cell type recognition of scRNA‐seq was used, with the reference transcriptomic datasets “Human Primary Cell Atlas”^[^ ^56^ ^]^ to infer the cell of origin of each of the single cells independently and identify cell types.

Differentially expressed genes (DEGs) were identified using the FindMarkers function (test. use = MAST) in Seurat.^[^ ^51^ ^]^ *p* value < 0.05 and |log_2_foldchange| > 0.58 was set as the threshold for significantly differential expression. GO enrichment and KEGG pathway enrichment analysis of DEGs were respectively performed using R package clusterProfiler^[^ ^57^ ^]^ based on the hypergeometric distribution.

The sequencing and bioinformatics analysis were performed by OE Biotech Co., Ltd. (Shanghai, China).

### Pseudotime Analysis

The developmental pseudotime was determined with the Monocle2 package^[^ ^58^ ^]^ (version 2.9.0). The raw count was first converted from the Seurat object into the CellDataSet object with the importCDS function in Monocle. The differentialGeneTest function of the Monocle2 package was used to select ordering genes (*q* val < 0.01) which were likely to be informative in the ordering of cells along the pseudotime trajectory. The dimensional reduction clustering analysis was performed with the reduceDimension function, followed by trajectory inference with the orderCells function using default parameters. Gene expression was plotted with the plot_genes_in_pseudotime function to track changes over pseudo‐time.

### scVelo Analysis

To perform the RNA velocity analysis, the spliced reads and unspliced reads were recounted using the Python script velocity. Py^[^ ^59^ ^]^(https://github.com/velocyto‐team/velocyto.py) on the Cell Ranger output folder. The likelihood‐based dynamical model and the velocity graph were constructed with scVelo^[^ ^60^ ^]^(https://scvelo.readthedocs.io/), to calculate the RNA velocities (rates of transcription, splicing, and degradation) of the single cells. Onto the UMAP embedding found in Seurat, the velocity fields were projected.

### Slingshot Analysis

Slingshot^[^ ^61^ ^]^ could provide the lineage structure and pseudotime variables inferences for cells along each lineage. The slingshot analysis was performed by slingshot R package (version 1.8.0). Initially, the dimensionally reduced Seurat object was transformed into a SingleCellExperiment object by as.SingleCellExperiment function. Then, the FitGAM function in the tradeSeq package^[^ ^62^ ^]^ (version 1.4.0) was used to fit the nonlinear function between gene expression and pseudotime using a negative binomial generalized additive model (NB‐GAM). By using the associationTest function, the top 100 genes whose expression levels significantly differed from pseudotime were chosen to create a heatmap.

### SCENIC Analysis

The SCENIC analysis was run using the motifs database for RcisTarget and GRNboost (SCENIC^[^ ^63^ ^]^ version 1.1.2.2, which corresponds to RcisTarget 1.2.1 and AUCell 1.4.1) with the default parameters. In detail, transcription factor (TF) binding motifs over‐represented on a gene list with the RcisTarget package were identified. The activity of each group of regulons in each cell was scored by the AUCell package.

To evaluate the cell type specificity of each predicted regulon, the regulon specificity score (RSS) was calculated which was based on the Jensen‐Shannon divergence (JSD), a measure of the similarity between two probability distributions. Specifically, the JSD (Jensen‐Shannon divergence) was calculated between each vector of binary regulon activity overlaps with the assignment of cells to a specific cell type.^[^ ^64^ ^]^ The connection specificity index (CSI) for all regulons was calculated with the scFunctions (https://github.com/FloWuenne/scFunctions/) package.

### Scoring of Biological Processes

Individual cells were scored for their expression of gene signatures representing certain biological functions. For all signatures except neutrophil aging, functional scores were defined as the average normalized expression of corresponding genes. Aging score and maturation score were calculated as previously described.^[^ ^28^ ^]^ Granule signatures,^[^ ^65^ ^]^ NADPH oxidase signatures,^[^ ^66^ ^]^ and NET formation signatures^[^ ^30^ ^]^ were from previous studies. Glycolysis signatures were from the Reactome Pathway Database (R‐MMU‐70171). Other functional signatures were derived from the Gene Ontology database.^[^ ^67^ ^]^ For instance, to access the phagocytosis function at the transcript level, a phagocytosis score was determined by calculating the average expression of genes in the Gene Ontology term “phagocytosis, engulfment” (GO: 0 006911). Similarly, the chemotaxis score was calculated by GO:00 30593 and the neutrophil activation score was calculated by GO:00 42119. The full gene list was provided in Table S5 (Supporting Information).

The AddModuleScore function in the Seurat R package was used to define the above. Specifically, the AddModuleScore function calculated the average expression levels of each program (cluster) on a single‐cell level, subtracted by the aggregated expression of control feature sets. All analyzed features were binned based on averaged expression, and the control features were randomly selected from each bin.

### Neutrophil Activation

To investigate neutrophil differentiation and NET formation, neutrophils isolated from the blood of patients with MI/RI and healthy donors were stimulated by ultrapure LPS (10 mg mL^−1^; L2630, Sigma) or Phorbol myristate acetate (PMA, 50 nM; P8139, Sigma) for 2 h as previously described.^[^ ^68^, ^69^, ^70^ ^]^ For the inhibitors study, neutrophils were treated with GSK484 (50 nm),^[^ ^31^ ^]^ DB2313 (660 nM),^[^ ^34^ ^]^ or vehicle (dimethyl sulfoxide, 1% v/v) 1 h before stimulation as described above.

### Immunofluorescence Staining and Confocal Microscopy

Neutrophils were attached to Confocal dishes coated with a poly‐L‐lysine (0.1 mg mL^−1^) solution (C0313, Beyotime) for 4 h and then stimulated as described before. Then, neutrophils were fixed with 4% paraformaldehyde (4%) for 30 min, blocked with 5% bovine serum albumin, and permeabilized with 0.3% Triton for 1 h. For NETs staining, the neutrophils were stained with the following antibodies: 1) rabbit anti‐citrullinated histone H3 (citH3, 1:2000; ab281584, Abcam) and goat anti‐rabbit IgG Alexa Fluor 488 (1:500; ab150077, Abcam); 2) Alexa Fluor 594‐conjugated anti‐Myeloperoxidase (MPO, 1:250; NB100‐64803AF594, Novus). For neutrophil differentiation, the neutrophils were stained with the following antibodies: 1) rabbit anti‐IFIT1 (1:200; ab236256, Abcam) and goat anti‐rabbit IgG Alexa Fluor 488 (1:500; ab150077, Abcam); 2) Alexa Fluor 647‐conjugated anti‐MMP9 (1:250; NBP2‐59699AF647, Novus). The nuclei were stained with 4′,6‐diamidino‐2‐phenylindole dihydrochloride (DAPI; C1005, Beyotime). Images were acquired using a confocal microscope (Olympus FV3000, Japan).

### Animals Study Design and Establishment of MI/RI Models

Wild‐type C57BL/6J mice aged 6–8 weeks were purchased from Shanghai Jiesijie Laboratory Animal Center (Shanghai, China). Gender‐matched mice were randomly divided into the saline group, GSK484 treated group, and DB2313 treated group. GSK484‐treated mice were injected intraperitoneally with 4mg kg^−1^ GSK484 (S7803, Selleck) per mouse as previously described.^[^ ^71^, ^72^ ^]^ DB2313‐treated mice were injected intraperitoneally with 17mg kg^−1^ DB2313 (HY‐124629, MedChemExpress) per mouse as previously described.^[^ ^34^ ^]^ Control group mice were injected intraperitoneally with 200 µL saline. The mice were injected with treatment or saline immediately after ligation, simulating clinical patients receiving medication treatment before PCI surgery in the clinic. For the tissue remodeling study, the mice were injected with treatment or saline immediately after ligation, 12, 36, and 72 h after MI/RI.

C57BL/6 mice carrying the floxed *Cst7* allele, obtained from Cyagen Biosciences, were crossed to mice carrying the S100A8‐Cre‐EGFP from Jackson Laboratory (Bar Harbor, ME). Sex and age‐matched mice were genotyped for *Cst7* ^fl/fl^ and S100A8‐Cre, and littermate *Cst7* ^fl/fl^ and *Cst7* ^CKO^ mice were used for all experiments, with the average age being 6–8 weeks.

MI/RI was performed as described previously.^[^ ^73^ ^]^ After being anesthetized with 2% isoflurane inhalation, the heart was exposed and the left anterior descending coronary artery was ligated using a 6.0 silk suture slipknot. After 45 min of occlusion, reperfusion was induced by the release of the slipknot. To fit the sampling time of human samples, mice were selected for surgery at zeitgeber time 10, and samples were taken 12 h after surgery. Due to the fact that the human control sample did not undergo sham surgery, the baseline control was chosen for the mouse control instead of the sham control.

All animal experiments were conducted following the animal welfare guidelines and approved by the Animal Care and Use Committee (Zhongshan Hospital, Fudan University, approval number: 2023–196). The animals were housed under pathogen‐free conditions and received care following the policies and accreditation standards of the ARRVE guidelines.

### Echocardiography

Mice were anesthetized with 2% isoflurane inhalation 12 h (or 7 days for tissue remodeling study) after surgery and subjected to transthoracic echocardiography (VeVo 2100 Imaging System; VisualSonics, Toronto, ON, Canada). The body temperatures of mice were maintained at 36.9–37.3 °C, while the heart rates of mice were maintained at 400–550 bpm. Using Echocardiographic M‐mode tracings records, at least six cardiac cycles were used to calculate left ventricular ejection fraction (LVEF) and left ventricular fractional shortening (LVFS).

### TTC‐Evans Blue Analysis

After reperfusion in mice, the left anterior descending artery was ligated again, and 1% Evans blue dye was injected into the aorta. Then, the heart was quickly removed and rinsed with 0.9% physiological saline. After removing the atria, cut the ventricles into 4–5 evenly thick slices from the apex to the base of the heart. Slices were incubated at 37 °C in 1% TTC (phosphate buffered saline) for 30 min, and then fixed in 10% formalin solution. The area not stained by TTC is the infarct area (IR, white), the area not stained by Evans blue is area at risk (AAR, red), and the non‐ischemic area is blue.

### Flow Cytometry

For clustering of neutrophils isolated from the blood of humans, after being washed with PBS twice, cells were blocked with Human BD Fc Block (Fc1.3216; 564 219, BD Pharmingen) on ice for 5 min. APC/cy7‐conjugated anti‐CD45 (557 833, BD Pharmingen), FITC‐conjugated anti‐CD66b (60 086, Stemcell), PE‐conjugated anti‐CD16 (60 041, Stemcell) were added and incubated for 30 min at 4 °C in the dark. Cells were washed with PBS twice, fixed, and permeabilized with Cytofix/Cytoperm Soln Kit (554 714, BD Pharmingen) according to the manufacturer's instructions. Then cells were stained with APC‐conjugated anti‐MMP9 (NBP2‐59699AF647, Novus) and PE/cy7‐conjugated anti‐IFIT1 (NBP2‐71005PECY7, Novus) antibodies for 30 min at 4 °C. Cells were then washed with PBS and resuspended in 1 mL stain buffer and ready for FACS study.

For the measure of ROS production and phagocytic ability of human neutrophils, cell suspensions were incubated for 30 min at 37 °C with the ROS‐sensitive probe Dihydrorhodamine‐123 (DHR123, ThermoFisher Scientific D23806, final concentration 1/200) or 10 µg mL^−1^ of fluorescent E. Coli bioparticles (Molecular Probes pHrodo Green E. coli BioParticles conjugates, ThermoFisher Scientific P35366), while negative control cells were incubated at 4 °C. After incubation, cells were immediately acquired for the FACS study.

For mice undergoing MI/RI, 12 h after surgery, hearts of mice were harvested, weighed, carefully cut into small pieces with fine scissors, and digested with type II collagenase (1.5 mg mL^−1^; LS004202, Worthington Biochemical Corporation), elastase (0.25 mg mL^−1^; LS002274, Worthington Biochemical Corporation), and DNase I (0.5 mg mL^−1^; LS002145, Worthington Biochemical Corporation) for 30 min at 37 °C into single cells suspension as previously described.^[^ ^74^ ^]^ After filtration by a 70 µm filter, the single cell suspension was lysed with Red Blood Cell Lysis Buffer (20110, Stemcell) to completely remove red blood cells. The blood of mice was collected in an EDTA anticoagulant tube and lysed with Red Blood Cell Lysis Buffer (20110, Stemcell) to completely remove red blood cells. After counting, cells were blocked with rat anti‐mouse CD16/CD32 antibody (553141, BD Pharmingen) on ice for 5 min. APC/cy7‐conjugated anti‐CD45 (557659, BD Pharmingen), FITC‐conjugated anti‐Ly6G (127606, Biolegend), PE‐conjugated anti‐CD11b (552850, BD Pharmingen) were added and incubated for 30 min at 4 °C in the dark. Cells were washed with PBS twice, fixed, and permeabilized with Cytofix/Cytoperm Soln Kit (554 714, BD Pharmingen) according to the manufacturer's instructions. Then cells were stained with APC‐conjugated anti‐MMP9 (NBP2‐59699AF647, Novus) and PE/cy7‐conjugated anti‐IFIT1 (NBP2‐71005PECY7, Novus) antibodies for 30 min at 4 °C. Cells were then washed with PBS and resuspended in 1 mL stain buffer and ready for FACS study.

The cell suspension was acquired on BD FACSAriaIII CellSorting System (BD Bio), and the data were analyzed by FlowJo v.9.5.2 software.

### Immunofluorescence Analysis of Mice Heart Sections

Mice were sacrificed 12 h after surgery and hearts were fixed with 4% paraformaldehyde. Paraffin sections were dewaxed in water by using xylene and alcohol, and then repaired in a microwave oven using an antigen repair buffer with EDTA (pH 8.0), before washing with PBS (pH 7.4). A histochemical pen was used to draw around the tissue, a self‐fluorescence quenching agent was added for 5 min, and the tissue was rinsed with water for 10 min. BSA was added for 30 min. The first type of antibody was added and incubated overnight at 4 °C in a wet box. After slightly drying the slices, drop the corresponding HRP‐labeled secondary antibody into the circle and incubate at room temperature for 50 min. After slightly drying the slices, add TSA dropwise into the circle and incubate at room temperature in the dark for 10 min. After incubation, the slides were placed in TBST and washed 3 times on a decolorization shaker, each time for 5 min. Place the repair box filled with repair solution in a microwave oven at high heat for 6 min, and place tissue slices in the repair box at low heat for 15 min to elution antibody. Repeat the above steps (change to another fluorescent dye) for the second round of labeling. After slightly drying the slices, add DAPI (C1005, Beyotime) staining solution dropwise into the circle and incubate at room temperature in the dark for 10 min. The sections were washed using PBS (pH 7.4) and sealed with anti‐fluorescence quenching sealing tablets (S2110, Solarbio, BJS CHN) before storing at 4 °C in a light‐proof box. The stained slides were photographed with a confocal microscope (Olympus FV3000, Japan).

For NETs staining, the neutrophils were stained with the following antibodies: (1) anti‐Ly6G antibody (1:1000; GB11229, Servicebio) and TYR‐570 Plus fluorescent dye (RC002, Recordbio); (2) anti‐MPO antibody (1:1000; GB11224, Servicebio) and TYR‐520 Plus fluorescent dye (RC001, Recordbio). For neutrophil differentiation, the neutrophils were stained with the following antibodies: (1) anti‐Ly6G antibody (1:1000; GB11229, Servicebio) and TYR‐570 Plus fluorescent dye (RC002, Recordbio); (2) rabbit anti‐IFIT1 antibody (1:200; ab236256, Abcam) and TYR‐520 Plus fluorescent dye (RC001, Recordbio).

### Masson's Trichrome Staining

Mice were sacrificed 7 days after surgery and hearts were fixed with 4% paraformaldehyde. Masson's trichrome staining was used to evaluate the extent of perivascular and interstitial collagen in the heart tissues. The index chosen for the measurement of collagen was the collagen volume fraction. By optical microscopy, the collagen volume fraction was verified using ImageJ software. Excluding endocardial collagen, the perivascular and interstitial collagen was calculated as the area occupied by the blue‐dyed tissue, divided by the total myocardial area under direct vision.

### NET Quantification (MPO/DNA Assay)

This procedure was performed as previously described.^[^ ^75^, ^76^ ^]^ Briefly, samples were incubated in the antibody‐coated Plate of MPO enzyme‐linked immunosorbent assay kit (ELISA, RK00313 for humans, RK00385 for mice, ABclonal) for 2 h. According to the manufacturer's instructions, the amount of DNA bound to the enzyme was quantified using the Quant‐iT PicoGreen dsDNA Reagent and Kit (P758, Invitrogen). The fluorescence intensity (excitation at 480 nm and emission at 520 nm) was quantified using Varioskan^TM^ LUX (Thermofisher).

### Enzyme‐Linked Immunosorbent Assay

Anesthetized mice's blood was collected 12 h after MI/RI, and after totally coagulated for 30 min at room temperature, the blood was centrifugated at 1500 g for 20 min and the serum was collected. Levels of human MMP9 and mouse MMP9 were determined by enzyme‐linked immunosorbent assay (ELISA; RK00217 and RK00187, Abclonal).

### Quantitative Real‐Time PCR

Total mRNA was extracted by Trizol reagent (Invitrogen, Carlsbad, CA, USA) and the mRNA was reversely transcribed to cDNA using a reverse transcription kit (TaKaRa Bio, Osaka, Japan). Quantitative real‐time PCR was performed using qPCR SYBR Green Master Mix (Yeasen Biotechnology, China). The comparative CT (2^‐ΔΔCT^) method was used for data analysis as previously described.^[^ ^77^ ^]^ The sequences of real‐time qPCR primers were listed in Table S10 (Supporting Information).

### Western Blot Assay

Total proteins were collected using RIPA lysis buffer (P0013C, Beyotime). Separated on 10%–15% SDS‐polyacrylamide gels by electrophoresis, the protein was transferred to polyvinylidene difluoride membranes. After being blocked with 5% bovine serum albumin in TBST for 1 h at room temperature, the membrane was incubated with the indicated primary antibodies at 4 °C overnight. After being washed in TBST for 5 min 3 times, the membrane was incubated with HRP‐conjugated secondary antibodies for 1 h at room temperature and washed in TBST for 5 min 3 times again. Immobilon western chemiluminescent HRP substrate (Millipore, Billerica, MA) was used for detection, and the images were obtained and analyzed using Image Lab 3.0 and ImageJ 1.8.0. The intensity of the β‐actin band was used as a loading control. Used primary antibodies were: MMP9 (10375‐2‐AP, Proteintech), β‐actin (KC‐5A08, KANGCHEN Biotech), citH3 (ab281584, Abcam), PADI4 (A16188, Abclonal), and CST7 (A8164, Abclonal).

### Dual‐Luciferase Reporter Assay and ChIP‐qPCR Assay

The 293T cells were purchased from ZQXZbio with STR profiling. The 293T cells were incubated in DMEM supplemented with 10% fetal bovine serum and 1% penicillin‐streptomycin at 37 °C with 5% CO_2_. 293T cells were suspended and seeded at the appropriate density in 24‐well plates. When the number of cells reached 60%−70%, a Dual‐Luciferase reporter assay was performed to explore the binding relationship between SPI1 and *CST7* promoter. The full *CST7* promoter region was constructed into the pGL4.14‐basic vector, and SPI1 was constructed into the pcDNA3.1–3x flag vector (Hanbio, China). The firefly luciferase plasmids h‐*CST7* (Full) or pGL3‐Basic (NC) were co‐transfected with SPI1 eukaryotic expression vectors (containing h‐SPI1 or pc‐DNA3.1–3x flag) and the pRL‐TK renilla luciferase vector (internal reference) into 293T cells. After 48 h, cell lysates were extracted, and firefly and Renilla luciferase activities were also detected using the Dual‐Luciferase Reporter Assay System (PROMEGA). The luminescence signal was detected using a multifunctional microplate reader (Varioskan LUX, Thermofisher). The experiment was repeated twice.

Human blood neutrophils were collected for ChIP‐qPCR assay. The ChIP assay was conducted according to the instruction manual (RK20258, Abclonal). The SPI1 antibody used in this assay was ChIP grade (2266, CST). The sequences of qPCR primers were listed in Table S10 (Supporting Information).

### Thioglycollate‐Induced Peritoneal Neutrophil Isolation

After intraperitoneal injection of 1.5 mL 3% Thioglycollate (Difco, 0236‐17‐7, suspend 30 g of the powder in 1 L of purified water, followed by 121 °C, 15 min autoclave; after cooling, store in a dark place at 4 °C) overnight, mice were anesthetized and abdominal infiltrates were collected with 10 mL PBS wash. Cells are centrifuged and resuspended in 45% Percoll and applied to the Percoll gradient column (81% + 62% percoll gradient) for neutrophil purification. Neutrophils are collected from the column after centrifuge and ready for use.

### RNA Sequencing

Total RNA was extracted and RNA purity, quantification, and integrity were assessed. Then the libraries were constructed using VAHTS Universal V6 RNA‐seq Library Prep Kit according to the manufacturer's instructions. The transcriptome sequencing and analysis were conducted by OE Biotech Co., Ltd. (Shanghai, China).

The libraries were sequenced on a Illumina Novaseq 6000 platform and 150 bp paired‐end reads were generated. Raw reads of fastq format were first processed using fastp and the low‐quality reads were removed to obtain the clean reads.T. The clean reads were mapped to the reference genome using HISAT2. FPKM of each gene was calculated and the read counts of each gene were obtained by HTSeq‐count. PCA analysis was performed using R (v 3.2.0) to evaluate the biological duplication of samples.

Differential expression analysis was performed using the DESeq2. Q value < 0.05 and foldchange > 2 or foldchange < 0.5 were set as the threshold for significantly differential expression genes (DEGs). Hierarchical cluster analysis of DEGs was performed using R (v 3.2.0) to demonstrate the expression pattern of genes in different groups and samples. The radar map of the top 30 genes was drawn to show the expression of up‐regulated or down‐regulated DEGs using an *R* packet grader.

### Statistical Analysis

Due to the long time span in sampling of human samples, we normalized neutrophil subset proportions from MI/RI patients with healthy donors from the same batch. All data were expressed as median with interquartile range (25th–75th percentiles) or mean ± standard error of the mean (SEM). Shapiro‐Wilk and q‐q‐plots were used for the normality test, and *p* > 0.05 on the test indicated that the data were approximately normally distributed. Brown‐Forsythe test was used for testing the homogeneity of variances, and *p* > 0.05 on the test indicated that the data in different groups fitted the homogeneity of variances. The normally distributed data with homogeneity of variances were compared using unpaired Student's *t*‐test (2 groups) or one‐way ANOVA (> 2 groups), and the Tukey‐Kramer test was used for multiple comparisons. If the data failed to meet the standard of normal distribution, the Mann‐Whitney test (2 groups) or Kruskal‐Wallis's test (> 2 groups) was used for the non‐parametric test, and Dunn's test was used for multiple comparisons. If the data failed to meet the standard of homogeneity of variances, the unpaired Student's *t*‐test with Welch's correction (2 groups) or Brown‐Forsythe test (> 2 groups) was used, and Dunnett's T3 multiple comparisons test was used for multiple comparisons. Linear regression and Pearson correlation analysis were used to analyze the relationship between normally distributed continuous variables. Linear regression and Spearman correlation analysis were used to analyze the relationship between continuous variables that failed to meet the standard of normal distribution. Tests used for each group of statistics were displayed in legends. Two‐sided *p *< 0.05 was considered statistically significant. Statistical analyses were conducted using GraphPad Prism 8.4.2 software.

## Conflict of Interest

Patent applications are being filed based on the findings described here.

## Author Contributions

S.H. and F.Z. contributed equally to this work. J.C., S.H., W.T., and J.G. performed the methodology. S.H., J.C., and W.T. performed conceptualization. S.H., F.Z., J.W., J.Z., J.C., Y.L., Y.W., R.H., J.Q., C.L., H.Y., and Y.G. performed the investigation. S.H., J.C., and W.T. performed visualization. J.G., F.Z., J.C., J.Q., and Y.G. performed Funding acquisition. J.G., F.Z., J.C., and W.T. performed supervision. S.H., J.C., and W.T. wrote the original draft. J.G., F.Z., S.H., J.C., and W.T. wrote, reviewed and edited the original draft.

## Supporting information



Supporting Information

Supplementary Table

## Data Availability

The data that support the findings of this study are available from the corresponding author upon reasonable request.

## References

[1] X. Li , W. Ou , M. Xie , J. Yang , Q. Li , T. Li , Adv. Healthcare Mater. 2023, 12, e2300161. 10.1002/adhm.202300161 PMC11468948 36971662

[2] G. Heusch , Nat. Rev. Cardiol. 2020, 17, 773. 32620851 10.1038/s41569-020-0403-y

[3] D. J. Hausenloy , D. M. Yellon , Nat. Rev. Cardiol. 2016, 13, 193. 26843289 10.1038/nrcardio.2016.5

[4] J. Vinten‐Johansen , Cardiovasc. Res. 2004, 61, 481. 14962479 10.1016/j.cardiores.2003.10.011

[5] A. Margraf , C. A. Lowell , A. Zarbock , Blood 2022, 139, 2130. 34624098 10.1182/blood.2021012295 PMC9728535

[6] H. R. Thiam , S. L. Wong , D. D. Wagner , C. M. Waterman , Annu. Rev. Cell Dev. Biol. 2020, 36, 191. 32663035 10.1146/annurev-cellbio-020520-111016 PMC8499668

[7] D. Dabrowska , E. Jablonska , M. Garley , W. Ratajczak‐Wrona , A. Iwaniuk , Scand. J. Immunol. 2016, 84, 317. 27667737 10.1111/sji.12494

[8] V. Papayannopoulos , Nat. Rev. Immunol. 2018, 18, 134. 28990587 10.1038/nri.2017.105

[9] K. Eghbalzadeh , L. Georgi , T. Louis , H. Zhao , U. Keser , C. Weber , M. Mollenhauer , A. Conforti , T. Wahlers , A. Paunel‐Gorgulu , Front. Immunol. 2019, 10, 2313. 31632398 10.3389/fimmu.2019.02313 PMC6779806

[10] Y. W. Li , S. X. Chen , Y. Yang , Z. H. Zhang , W. B. Zhou , Y. N. Huang , Z. Q. Huang , J. Q. He , T. F. Chen , J. F. Wang , Z. Y. Liu , Y. X. Chen , Cardiovasc. Drugs Ther. 2024, 38, 31. 35900652 10.1007/s10557-022-07326-y

[11] K. Yang , R. Gao , H. Chen , J. Hu , P. Zhang , X. Wei , J. Shi , Y. Chen , L. Zhang , J. Chen , Y. Lyu , Z. Dong , W. Wei , K. Hu , Y. Guo , J. Ge , A. Sun , Eur. Heart J. 2024, 45, 1662. 38666340 10.1093/eurheartj/ehae205 PMC11089336

[12] Z. Zhang , S. Ding , Z. Wang , X. Zhu , Z. Zhou , W. Zhang , X. Yang , J. Ge , Acta Pharm. Sin. B 2022, 12, 1840. 35847488 10.1016/j.apsb.2021.10.016 PMC9279636

[13] K. Ley , H. M. Hoffman , P. Kubes , M. A. Cassatella , A. Zychlinsky , C. C. Hedrick , S. D. Catz , Sci. Immunol. 2018, 3. 10.1126/sciimmunol.aat4579 30530726

[14] L. G. Ng , R. Ostuni , A. Hidalgo , Nat. Rev. Immunol. 2019, 19, 255. 30816340 10.1038/s41577-019-0141-8

[15] L. Yvan‐Charvet , L. G. Ng , Trends Immunol. 2019, 40, 598. 31256783 10.1016/j.it.2019.05.004

[16] K. Sun , Y. Y. Li , J. Jin , Signal Transduct. Target Ther. 2021, 6, 79. 33612829 10.1038/s41392-020-00455-6 PMC7897720

[17] J. G. Rurik , H. Aghajanian , J. A. Epstein , Circ. Res. 2021, 128, 1766. 34043424 10.1161/CIRCRESAHA.121.318005 PMC8171813

[18] M. Phillipson , P. Kubes , Trends Immunol. 2019, 40, 635. 31160208 10.1016/j.it.2019.05.001

[19] L. Adlung , I. Amit , Nat. Rev. Immunol. 2018, 18, 597. 30078033 10.1038/s41577-018-0050-2

[20] M. J. T. Stubbington , O. Rozenblatt‐Rosen , A. Regev , S. A. Teichmann , Science 2017, 358, 58. 28983043 10.1126/science.aan6828 PMC5654495

[21] E. Vafadarnejad , G. Rizzo , L. Krampert , P. Arampatzi , A. P. Arias‐Loza , Y. Nazzal , A. Rizakou , T. Knochenhauer , S. R. Bandi , V. A. Nugroho , D. J. J. Schulz , M. Roesch , P. Alayrac , J. Vilar , J. S. Silvestre , A. Zernecke , A. E. Saliba , C. Cochain , Circ. Res. 2020, 127, e232. 32811295 10.1161/CIRCRESAHA.120.317200

[22] K. Jin , S. Gao , P. Yang , R. Guo , D. Li , Y. Zhang , X. Lu , G. Fan , X. Fan , Small Methods 2022, 6, e2100752. 35023642 10.1002/smtd.202100752

[23] D. M. Calcagno , C. Zhang , A. Toomu , K. Huang , V. K. Ninh , S. Miyamoto , A. D. Aguirre , Z. Fu , J. Heller Brown , K. R. King , J. Am. Heart Assoc. 2021, 10, e019019. 33525909 10.1161/JAHA.120.019019 PMC7955351

[24] Y. Dong , Z. Kang , Z. Zhang , Y. Zhang , H. Zhou , Y. Liu , X. Shuai , J. Li , L. Yin , X. Wang , Y. Ma , H. Fan , F. Jiang , Z. Lin , C. Ding , K. Yun Jin , A. Sarapultsev , F. Li , G. Zhang , T. Xie , C. Yin , X. Cheng , S. Luo , Y. Liu , D. Hu , Sci. Bull. 2024, 69, 949. 10.1016/j.scib.2024.02.003 38395651

[25] V. Rusinkevich , Y. Huang , Z. Y. Chen , W. Qiang , Y. G. Wang , Y. F. Shi , H. T. Yang , Acta Pharmacol. Sin. 2019, 40, 1168. 30858476 10.1038/s41401-018-0197-1 PMC6786364

[26] S. Chia , J. T. Nagurney , D. F. Brown , O. C. Raffel , F. Bamberg , F. Senatore , F. J. Wackers , I. K. Jang , Am. J. Cardiol. 2009, 103, 333. 19166685 10.1016/j.amjcard.2008.09.085

[27] L. Jiang , X. P. Li , Y. T. Dai , B. Chen , X. Q. Weng , S. M. Xiong , M. Zhang , J. Y. Huang , Z. Chen , S. J. Chen , Proc. Natl. Acad. Sci. U.S.A. 2020, 117, 20117. 32747558 10.1073/pnas.2003900117 PMC7443908

[28] X. Xie , Q. Shi , P. Wu , X. Zhang , H. Kambara , J. Su , H. Yu , S. Y. Park , R. Guo , Q. Ren , S. Zhang , Y. Xu , L. E. Silberstein , T. Cheng , F. Ma , C. Li , H. R. Luo , Nat. Immunol. 2020, 21, 1119. 32719519 10.1038/s41590-020-0736-z PMC7442692

[29] N. Borregaard , T. Herlin , J. Clin. Invest. 1982, 70, 550. 7107894 10.1172/JCI110647 PMC370256

[30] X. T. Shen , S. Z. Xie , J. Xu , L. Y. Yang , L. X. Qin , Front. Immunol. 2022, 13, 798022. 35432310 10.3389/fimmu.2022.798022 PMC9009150

[31] H. D. Lewis , J. Liddle , J. E. Coote , S. J. Atkinson , M. D. Barker , B. D. Bax , K. L. Bicker , R. P. Bingham , M. Campbell , Y. H. Chen , C. W. Chung , P. D. Craggs , R. P. Davis , D. Eberhard , G. Joberty , K. E. Lind , K. Locke , C. Maller , K. Martinod , C. Patten , O. Polyakova , C. E. Rise , M. Rudiger , R. J. Sheppard , D. J. Slade , P. Thomas , J. Thorpe , G. Yao , G. Drewes , D. D. Wagner , et al., Nat. Chem. Biol. 2015, 11, 189. 25622091 10.1038/nchembio.1735 PMC4397581

[32] J. Cedervall , A. Dragomir , F. Saupe , Y. Zhang , J. Arnlov , E. Larsson , A. Dimberg , A. Larsson , A. K. Olsson , Oncoimmunology 2017, 6, e1320009. 28919990 10.1080/2162402X.2017.1320009 PMC5593702

[33] Z. Ai , I. A. Udalova , J. Leukoc. Biol. 2020, 107, 419. 31951039 10.1002/JLB.1RU1219-504RR

[34] I. Antony‐Debre , A. Paul , J. Leite , K. Mitchell , H. M. Kim , L. A. Carvajal , T. I. Todorova , K. Huang , A. Kumar , A. A. Farahat , B. Bartholdy , S. R. Narayanagari , J. Chen , A. Ambesi‐Impiombato , A. A. Ferrando , I. Mantzaris , E. Gavathiotis , A. Verma , B. Will , D. W. Boykin , W. D. Wilson , G. M. Poon , U. Steidl , J. Clin. Invest. 2017, 127, 4297. 29083320 10.1172/JCI92504 PMC5707147

[35] S. Zhang , S. Zhao , Y. Qi , B. Li , H. Wang , Z. Pan , H. Xue , C. Jin , W. Qiu , Z. Chen , Q. Guo , Y. Fan , J. Xu , Z. Gao , S. Wang , X. Guo , L. Deng , S. Ni , F. Xue , J. Wang , R. Zhao , G. Li , Mol. Ther. Nucleic. Acids 2022, 27, 699. 35317283 10.1016/j.omtn.2021.12.035 PMC8905236

[36] A. J. Sawyer , M. Garand , D. Chaussabel , C. G. Feng , Front. Immunol. 2021, 12, 634119. 33868254 10.3389/fimmu.2021.634119 PMC8047108

[37] J. Albrengues , M. A. Shields , D. Ng , C. G. Park , A. Ambrico , M. E. Poindexter , P. Upadhyay , D. L. Uyeminami , A. Pommier , V. Kuttner , E. Bruzas , L. Maiorino , C. Bautista , E. M. Carmona , P. A. Gimotty , D. T. Fearon , K. Chang , S. K. Lyons , K. E. Pinkerton , L. C. Trotman , M. S. Goldberg , J. T. Yeh , M. Egeblad , Science 2018, 361. 10.1126/science.aao4227 PMC6777850 30262472

[38] S. Duarte , P. Matian , S. Ma , R. W. Busuttil , A. J. Coito , Am. J. Pathol. 2018, 188, 1820. 29870740 10.1016/j.ajpath.2018.05.002 PMC6099362

[39] T. Hamada , C. Fondevila , R. W. Busuttil , A. J. Coito , Hepatology 2008, 47, 186. 17880014 10.1002/hep.21922

[40] P. G. Jobin , G. S. Butler , C. M. Overall , Biochim. Biophys. Acta. Mol. Cell Res. 1864, 2017, 2043. 10.1016/j.bbamcr.2017.05.013 28526562

[41] S. K. Yadav , P. K. Mishra , Stem. Cells 2021, 39, 497. 33438302 10.1002/stem.3330 PMC9188476

[42] H. Hirai , P. Zhang , T. Dayaram , C. J. Hetherington , S. Mizuno , J. Imanishi , K. Akashi , D. G. Tenen , Nat. Immunol. 2006, 7, 732. 16751774 10.1038/ni1354

[43] M. G. Manz , S. Boettcher , Nat. Rev. Immunol. 2014, 14, 302. 24751955 10.1038/nri3660

[44] R. Grieshaber‐Bouyer , F. A. Radtke , P. Cunin , G. Stifano , A. Levescot , B. Vijaykumar , N. Nelson‐Maney , R. B. Blaustein , P. A. Monach , P. A. Nigrovic , C. ImmGen , Nat. Commun. 2021, 12, 2856. 34001893 10.1038/s41467-021-22973-9 PMC8129206

[45] C. Nerlov , T. Graf , Genes Dev. 1998, 12, 2403. 9694804 10.1101/gad.12.15.2403 PMC317050

[46] G. Li , W. Hao , W. Hu , Int. J. Mol. Med. 2020, 46, 1943. 33125129 10.3892/ijmm.2020.4763

[47] N. S. Hackert , F. A. Radtke , T. Exner , H. M. Lorenz , C. Muller‐Tidow , P. A. Nigrovic , G. Wabnitz , R. Grieshaber‐Bouyer , Nat. Commun. 2023, 14, 8133. 38065997 10.1038/s41467-023-43573-9 PMC10709367

[48] J. Fischer , C. Walter , A. Tonges , H. Aleth , M. J. C. Jordao , M. Leddin , V. Groning , T. Erdmann , G. Lenz , J. Roth , T. Vogl , M. Prinz , M. Dugas , I. D. Jacobsen , F. Rosenbauer , Nat. Immunol. 2019, 20, 546. 30911105 10.1038/s41590-019-0343-z

[49] M. Horckmans , L. Ring , J. Duchene , D. Santovito , M. J. Schloss , M. Drechsler , C. Weber , O. Soehnlein , S. Steffens , Eur. Heart J. 2017, 38, 187. 28158426 10.1093/eurheartj/ehw002

[50] A. Warnatsch , M. Ioannou , Q. Wang , V. Papayannopoulos , Science 2015, 349, 316. 26185250 10.1126/science.aaa8064 PMC4854322

[51] A. Butler , P. Hoffman , P. Smibert , E. Papalexi , R. Satija , Nat. Biotechnol. 2018, 36, 411. 29608179 10.1038/nbt.4096 PMC6700744

[52] C. S. McGinnis , L. M. Murrow , Z. J. Gartner , Cell Syst. 2019, 8, 329. 30954475 10.1016/j.cels.2019.03.003 PMC6853612

[53] E. Z. Macosko , A. Basu , R. Satija , J. Nemesh , K. Shekhar , M. Goldman , I. Tirosh , A. R. Bialas , N. Kamitaki , E. M. Martersteck , J. J. Trombetta , D. A. Weitz , J. R. Sanes , A. K. Shalek , A. Regev , S. A. McCarroll , Cell 2015, 161, 1202. 26000488 10.1016/j.cell.2015.05.002 PMC4481139

[54] L. Haghverdi , A. T. L. Lun , M. D. Morgan , J. C. Marioni , Nat. Biotechnol. 2018, 36, 421. 29608177 10.1038/nbt.4091 PMC6152897

[55] D. Aran , A. P. Looney , L. Liu , E. Wu , V. Fong , A. Hsu , S. Chak , R. P. Naikawadi , P. J. Wolters , A. R. Abate , A. J. Butte , M. Bhattacharya , Nat. Immunol. 2019, 20, 163. 30643263 10.1038/s41590-018-0276-y PMC6340744

[56] N. A. Mabbott , J. K. Baillie , H. Brown , T. C. Freeman , D. A. Hume , BMC Genomics 2013, 14, 632. 24053356 10.1186/1471-2164-14-632 PMC3849585

[57] T. Wu , E. Hu , S. Xu , M. Chen , P. Guo , Z. Dai , T. Feng , L. Zhou , W. Tang , L. Zhan , X. Fu , S. Liu , X. Bo , G. Yu , Innovation 2021, 2, 100141. 34557778 10.1016/j.xinn.2021.100141 PMC8454663

[58] C. Trapnell , D. Cacchiarelli , J. Grimsby , P. Pokharel , S. Li , M. Morse , N. J. Lennon , K. J. Livak , T. S. Mikkelsen , J. L. Rinn , Nat. Biotechnol. 2014, 32, 381. 24658644 10.1038/nbt.2859 PMC4122333

[59] G. L. Manno , R. Soldatov , A. Zeisel , E. Braun , H. Hochgerner , V. Petukhov , K. Lidschreiber , M. E. Kastriti , P. Lonnerberg , A. Furlan , J. Fan , L. E. Borm , Z. Liu , D. van Bruggen , J. Guo , X. He , R. Barker , E. Sundstrom , G. Castelo‐Branco , P. Cramer , I. Adameyko , S. Linnarsson , P. V. Kharchenko , Nature 2018, 560, 494. 30089906 10.1038/s41586-018-0414-6 PMC6130801

[60] V. Bergen , M. Lange , S. Peidli , F. A. Wolf , F. J. Theis , Nat. Biotechnol. 2020, 38, 1408. 32747759 10.1038/s41587-020-0591-3

[61] K. Street , D. Risso , R. B. Fletcher , D. Das , J. Ngai , N. Yosef , E. Purdom , S. Dudoit , BMC Genomics 2018, 19, 477. 29914354 10.1186/s12864-018-4772-0 PMC6007078

[62] K. Van den Berge , H. Roux de Bezieux , K. Street , W. Saelens , R. Cannoodt , Y. Saeys , S. Dudoit , L. Clement , Nat. Commun. 2020, 11, 1201. 32139671 10.1038/s41467-020-14766-3 PMC7058077

[63] S. Aibar , C. B. Gonzalez‐Blas , T. Moerman , V. A. Huynh‐Thu , H. Imrichova , G. Hulselmans , F. Rambow , J. C. Marine , P. Geurts , J. Aerts , J. van den Oord , Z. K. Atak , J. Wouters , S. Aerts , Nat. Methods 2017, 14, 1083. 28991892 10.1038/nmeth.4463 PMC5937676

[64] S. Suo , Q. Zhu , A. Saadatpour , L. Fei , G. Guo , G. C. Yuan , Cell Rep. 2018, 25, 1436. 30404000 10.1016/j.celrep.2018.10.045 PMC6281296

[65] J. B. Cowland , N. Borregaard , Immunol. Rev. 2016, 273, 11. 27558325 10.1111/imr.12440

[66] L. M. Henderson , J. B. Chappel , Biochim. Biophys. Acta 1996, 1273, 87. 8611594 10.1016/0005-2728(95)00140-9

[67] C. The Gene Ontology , Nucleic Acids Res. 2019, 47, D330. 30395331 10.1093/nar/gky1055 PMC6323945

[68] K. W. Chen , M. Monteleone , D. Boucher , G. Sollberger , D. Ramnath , N. D. Condon , J. B. von Pein , P. Broz , M. J. Sweet , K. Schroder , Sci. Immunol. 2018, 3. 10.1126/sciimmunol.aar6676 30143554

[69] Y. Xiao , M. Cong , J. Li , D. He , Q. Wu , P. Tian , Y. Wang , S. Yang , C. Liang , Y. Liang , J. Wen , Y. Liu , W. Luo , X. Lv , Y. He , D. D. Cheng , T. Zhou , W. Zhao , P. Zhang , X. Zhang , Y. Xiao , Y. Qian , H. Wang , Q. Gao , Q. C. Yang , Q. Yang , G. Hu , Cancer Cell 2021, 39, 423. 33450198 10.1016/j.ccell.2020.12.012

[70] C. M. S. Silva , C. W. S. Wanderley , F. P. Veras , F. Sonego , D. C. Nascimento , A. V. Goncalves , T. V. Martins , D. F. Colon , V. F. Borges , V. S. Brauer , L. E. A. Damasceno , K. P. Silva , J. E. Toller‐Kawahisa , S. S. Batah , A. L. J. Souza , V. S. Monteiro , A. E. R. Oliveira , P. B. Donate , D. Zoppi , M. C. Borges , F. Almeida , H. I. Nakaya , A. T. Fabro , T. M. Cunha , J. C. Alves‐Filho , D. S. Zamboni , F. Q. Cunha , Blood 2021, 138, 2702. 34407544 10.1182/blood.2021011525 PMC8703366

[71] L. Wei , X. Wang , M. Luo , H. Wang , H. Chen , C. Huang , Hum. Exp. Toxicol. 2021, 40, 1074. 33355008 10.1177/0960327120979028

[72] C. Yang , C. Song , Y. Liu , J. Qu , H. Li , W. Xiao , L. Kong , H. Ge , Y. Sun , W. Lv , Phytomedicine 2021, 90, 153635. 34229173 10.1016/j.phymed.2021.153635 PMC8213523

[73] H. Shi , Y. Gao , Z. Dong , J. Yang , R. Gao , X. Li , S. Zhang , L. Ma , X. Sun , Z. Wang , F. Zhang , K. Hu , A. Sun , J. Ge , Circ. Res. 2021, 129, 383. 34015941 10.1161/CIRCRESAHA.120.318629 PMC8291144

[74] D. Jia , H. Jiang , X. Weng , J. Wu , P. Bai , W. Yang , Z. Wang , K. Hu , A. Sun , J. Ge , Circ. Res. 2019, 124, 1323. 30832557 10.1161/CIRCRESAHA.118.314569

[75] D. F. Colon , C. W. Wanderley , M. Franchin , C. M. Silva , C. H. Hiroki , F. V. S. Castanheira , P. B. Donate , A. H. Lopes , L. C. Volpon , S. K. Kavaguti , V. F. Borges , C. A. Speck‐Hernandez , F. Ramalho , A. P. Carlotti , F. Carmona , J. C. Alves‐Filho , F. Y. Liew , F. Q. Cunha , Crit. Care 2019, 23, 113. 30961634 10.1186/s13054-019-2407-8 PMC6454713

[76] L. Luo , S. Zhang , Y. Wang , M. Rahman , I. Syk , E. Zhang , H. Thorlacius , Am. J. Physiol. Lung Cell Mol. Physiol. 2014, 307, L586. 25085626 10.1152/ajplung.00365.2013

[77] A. Kladi‐Skandali , D. C. Sideris , A. Scorilas , Clin. Chem. Lab. Med. 2018, 57, 276. 30325729 10.1515/cclm-2018-0272

