# Supplementary material for: MMP9High Neutrophils are Critical Mediators of Neutrophil Extracellular Traps Formation and Myocardial Ischemia/Reperfusion Injury
Source: Adv Sci (Weinh). 2025 Mar 28;12(21):2415205. doi: 10.1002/advs.202415205 (PMC12140383; doi:10.1002/advs.202415205)
Supplement: Supplementary file 1 — Supporting Information [file ADVS-12-2415205-s002.docx]

Supporting Information

MMP9^High^ Neutrophils Are Critical Mediators of Neutrophil Extracellular Traps Formation and Myocardial Ischemia/Reperfusion Injury

*Shiyu Hu*,^1,3,4,5,6†^ *Feng Zhang*,^1,3,4,5,6†*^ *Jingpu Wang*,^1,3,4,5,6^ *Jian Zhang*,^1,3,4,5,6^ *Chenguang Li*,^1,3,4,5,6^ *Yang Lyu*,^7^ *Yiwen Wang*,^1,3,4,5,6^ *Rong Huang*,^1,3,4,5,6^ *Yang Gao*,^1,3,4,5,6^ *Hongbo Yang*,^1,3,4,5,6^ *Juying Qian*,^1,3,4,5,6^ *Wenwen Tang*,^2*^ *Jiatian Cao*,^1,3,4,5,6*^ *Junbo Ge*^1,3,4,5,6*^

**Supporting Information**

Figure S1 to Figure S12

Table S1 to Table S10 (Table S2 to Table S6 and Table S8 to Table S10 were provided as a separate .xlsx file)

**Figure S1 To Figure S12**


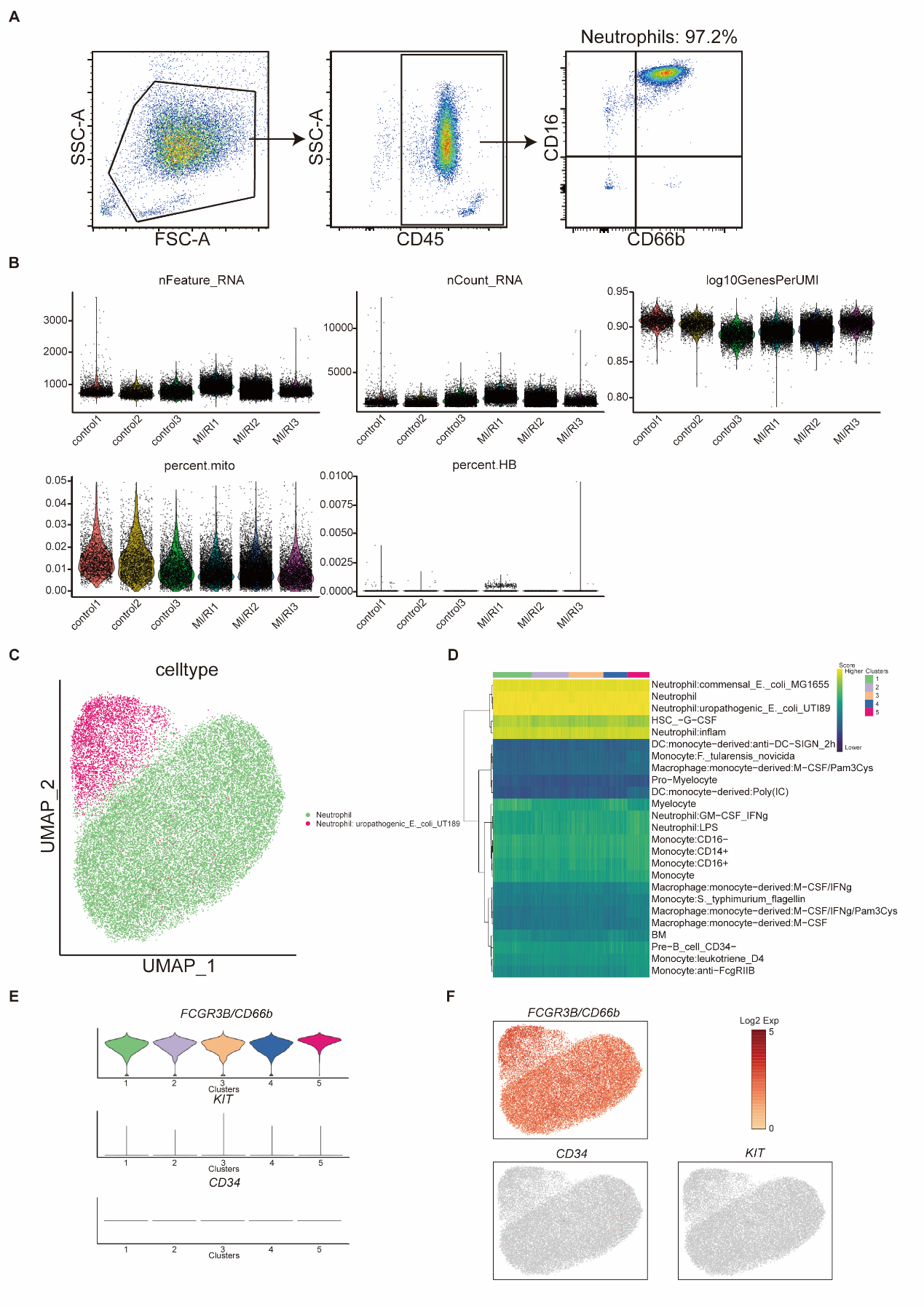


**Figure S1. Quality control of neutrophil purity, scRNA-seq, and cell type annotation.** **(A)** Flow cytometry of purity of neutrophils separation. **(B)** Quantitative gene quality control and filtration of low-quality cells. **(C)** Human HPCA reference single cell typing plot. **(D)** Human HPCA reference single cell typing heatmap. **(E)** Violin plots of specific surface markers of human neutrophil (*FCGR3B*/*CD66b*) and specific markers of granulocyte monocyte progenitor cells (*KIT* and *CD34*) for each cluster. **(F)** Uniform manifold approximation and projection (UMAP) representation of gene expression of *FCGR3B*/*CD66b*, *KIT*, and *CD34*.


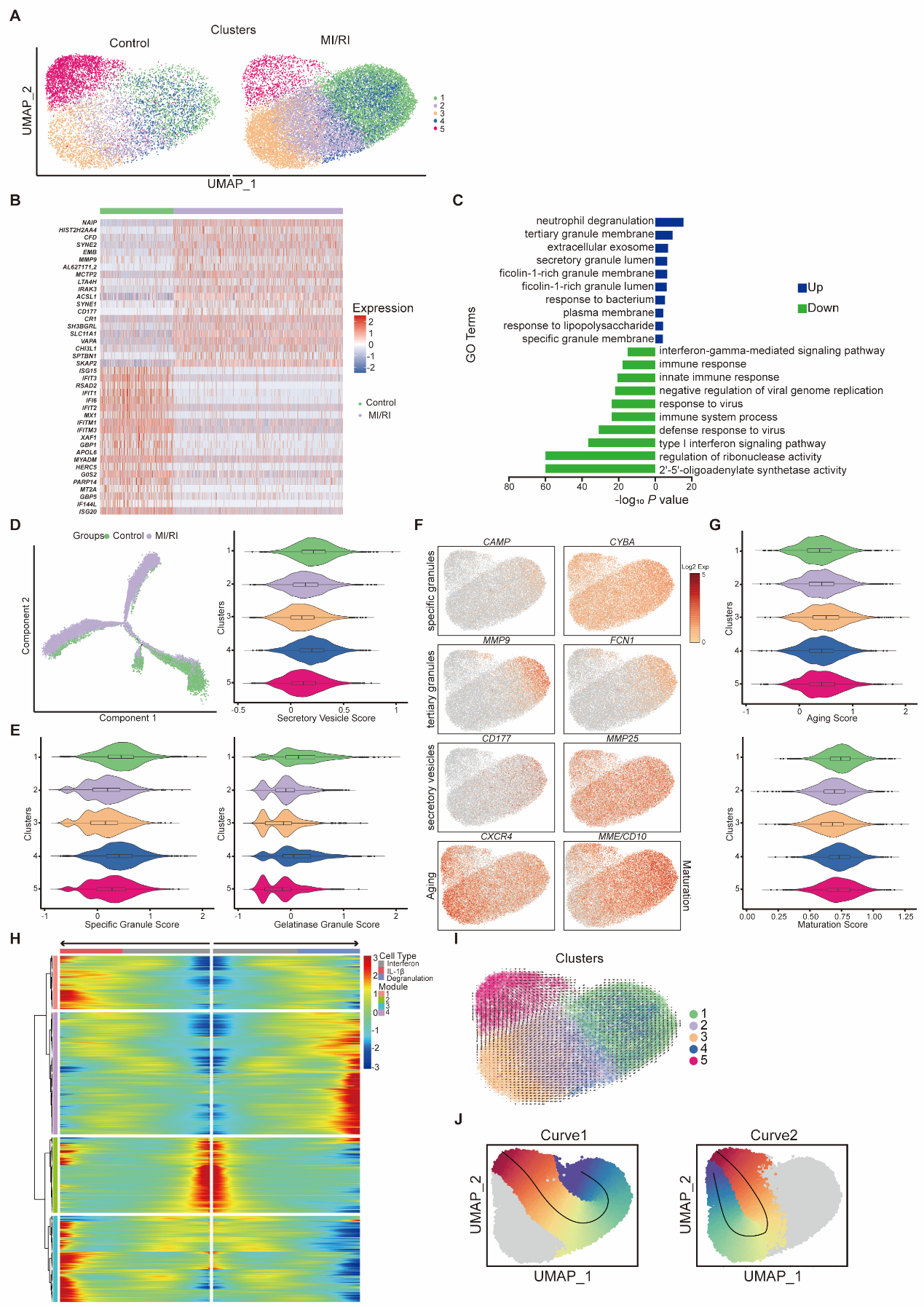


**Figure S2. Extended scRNA-seq analysis, neutrophil differentiation trajectories analysis, and functional analysis.** **(A)** UMAP representation of gene expression data in Clusters 1-5 with Seurat cluster assignment projected onto the UMAP plot split by group. **(B)** Heatmap of the top 20 DEGs associated with the MI/RI group versus the Control group. **(C)** Gene Ontology analysis of DEGs for the MI/RI group versus the Control group. **(D)** Monocle trajectories of neutrophils colored by group. Each dot represents a single cell. **(E)** Violin plot of specific granule scores, tertiary granule scores, and secretory vesicle scores for each cluster. **(F)** UMAP representation of gene expression of marker genes for specific granules (*CAMP* and *CYBA*), tertiary granules (*MMP9* and *FCN1*), secretory vesicles (*CD177* and *MMP25*), aging (*CXCR4*), and maturation (*MME/CD10*). **(G)** Violin plot of aging scores and maturation scores for each cluster. **(H)** Heatmap of gene expression variation according to brunchtime of pseudotime and marker genes in each module. **(I)** Velocity analysis revealing the origin and inter-relationship of neutrophil subpopulations. Velocity fields were projected onto the UMAP plot. **(J)** Slingshot analysis simulated Pseudotime.


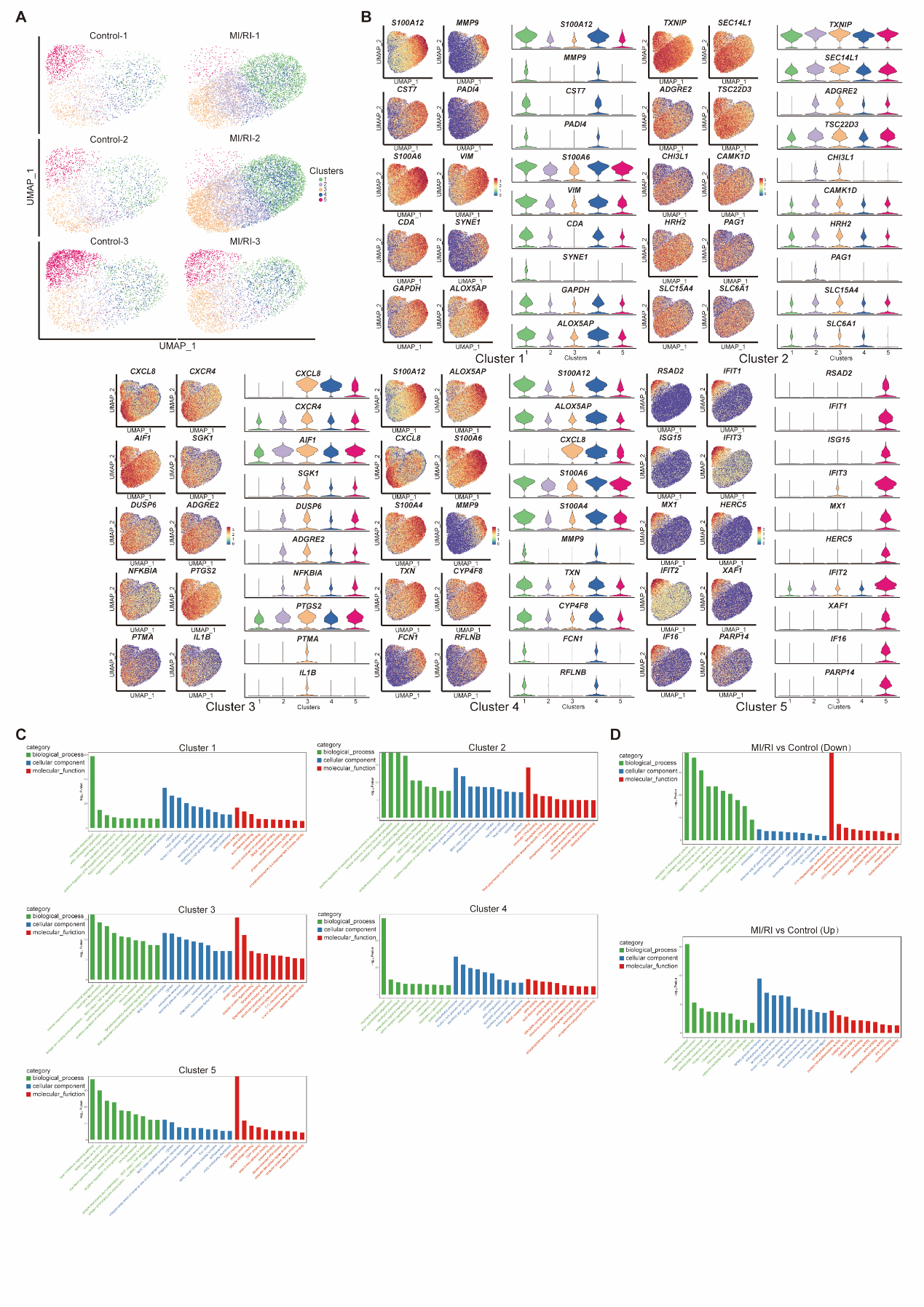


**Figure S3. Extended scRNA-seq analysis of neutrophils from the blood of MI/RI patients and healthy donors.** **(A)** UMAP representation of gene expression data in Clusters 1-5 split by sample. **(B)** UMAP representation and violin plot of marker gene of each cluster. **(C)** Gene Ontology analysis of DEGs for each of the 5 clusters. **(D)** Gene Ontology analysis of DEGs down-regulation (top) and up-regulation (bottom) for MI/RI group versus Control group.

**
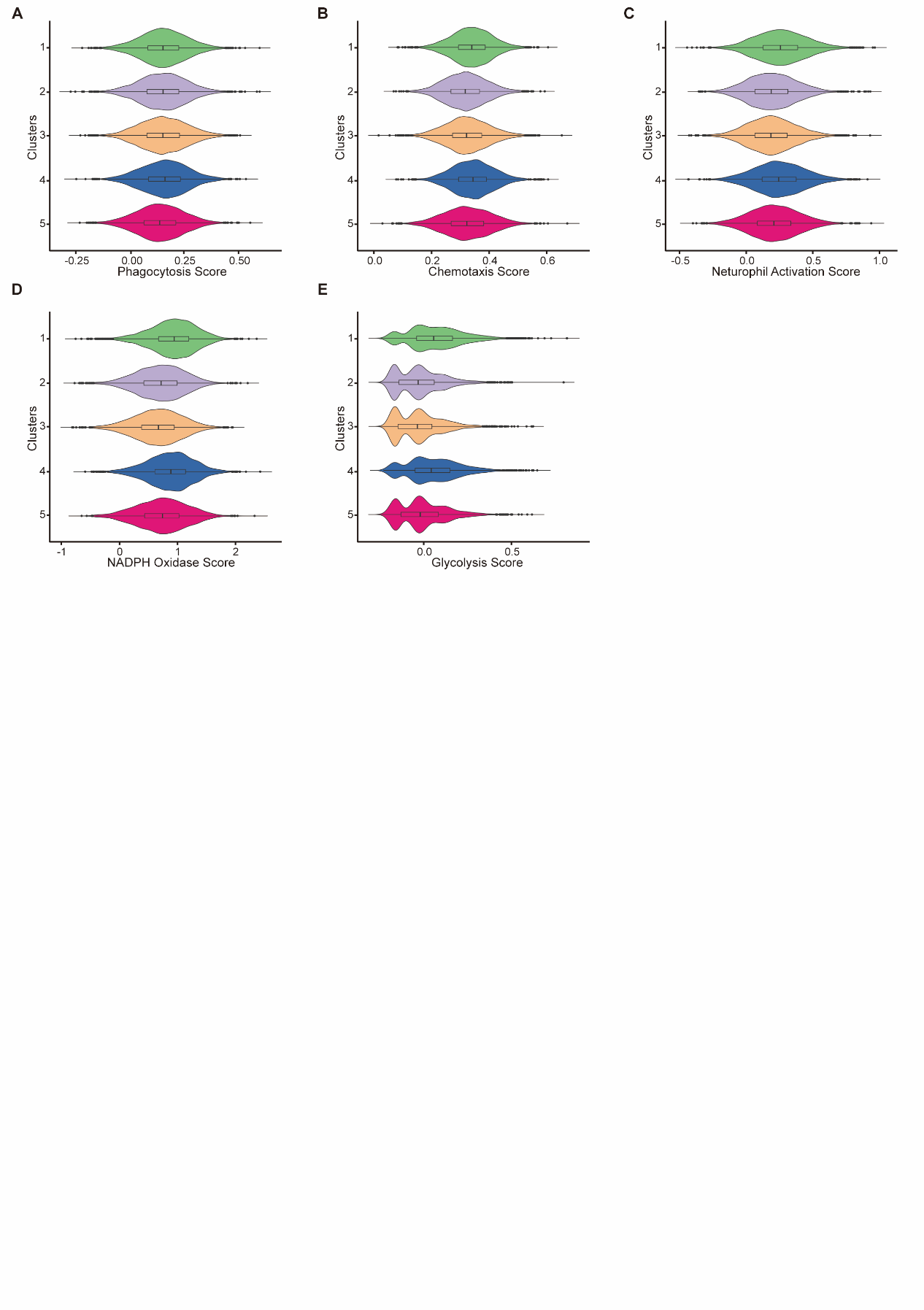
**

**Figure S4. Extended neutrophil cluster scoring.** **(A-E)** Violin plot of phagocytosis score (GO:0006911), chemotaxis score (GO:0030593), neutrophil activation score (GO:0042119), NADPH oxidase score, and Glycolysis score (Reactome Pathway Database #R-MMU-70171) for each cluster.


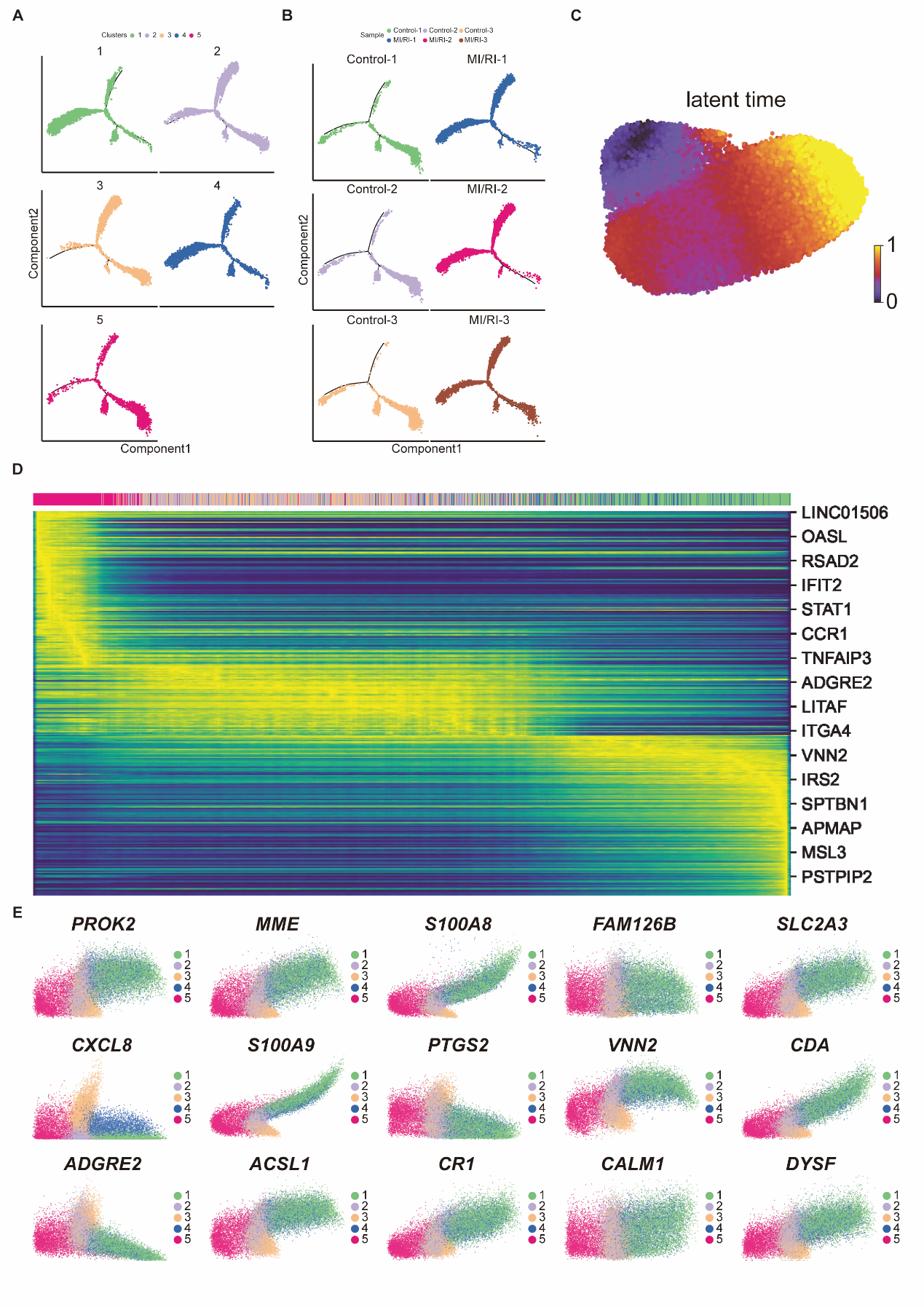


**Figure S5. Extended analysis of neutrophil clusters differentiation trajectories. (A)** Monocle trajectories of neutrophils for each cluster. **(B)** Monocle trajectories of neutrophils for each sample. **(C)** Latent time by dynamically projected onto the UMAP plot. **(D)** Heatmap of top 100 likelihood driving genes expression variation according to latent time. **(E)** Top 15 likelihood driving genes alone time grouped by clusters.


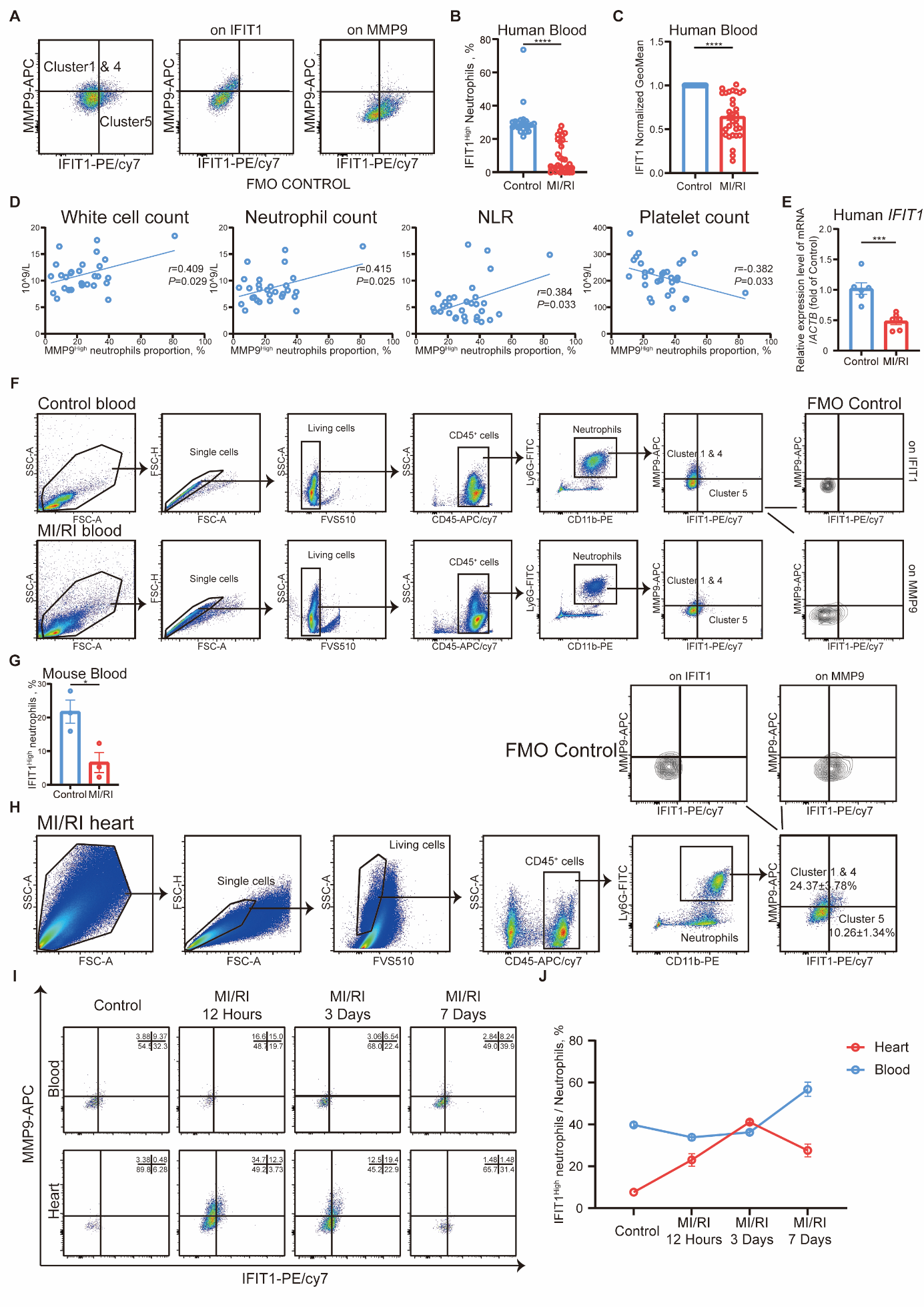


**Figure S6. Extended data of neutrophil clustering in human blood and the mouse model.** **(A)** Fluorescence minus one (FMO) controls (right) were used to control for spillover-related contribution to the background in MMP9-APC and IFIT1-PE/cy7 channel. **(B)** Proportion of IFIT1^High^ neutrophils (Mann-Whitney test) in healthy donors (*n*=22) and MI/RI patients (*n*=31). **(C)** Relative fluorescence density (one sample *t*-test) of IFIT1 in MI/RI patients (*n*=31) compared to healthy donors (*n*=22, normalized to 1 at same batch). **(D)** Spearman correlation analysis of MMP9^High^ neutrophils with white blood cell count, neutrophil count, NLR, and platelet count (*n*=29-31). **(E)** Relative mRNA levels of *IFIT1* (unpaired *t* test) of neutrophils (*n*=6). **(F)** FACS and staining strategy for mouse blood neutrophil (CD45^+^CD11b^+^Ly6G^+^), Clusters 1 & 4 (MMP9^High^), and Cluster 5 (IFIT1^High^). Fluorescence minus one (FMO) controls (right) were used to control for spillover-related contribution to the background in MMP9-APC and IFIT1-PE/cy7 channel. **(G)** Proportion of IFIT1^High^ neutrophils (unpaired *t* test) in the blood of control mice (*n*=3) and MI/RI mice (*n*=3). **(H)** FACS and staining strategy for mouse heart neutrophil (CD45^+^CD11b^+^Ly6G^+^), Clusters 1 & 4 (MMP9^High^), and Cluster 5 (IFIT1^High^). Fluorescence minus one (FMO) controls (top) were used to control for spillover-related contribution to the background in MMP9-APC and IFIT1-PE/cy7 channel. Proportion of MMP9^High^ neutrophils and IFIT1^High^ neutrophils were displayed as mean ± SEM (*n*=4). **(I)** Representative FACs images of neutrophil clustering of neutrophil from the blood and heart of mice along time after MI/RI. **(J)** Proportion of IFIT1^High^ neutrophils in neutrophils from blood and heart of mice along time after MI/RI (*n*=3-6 each). All data was displayed as median with interquartile range or mean ± SEM. * *p* < 0.05; *** *p* < 0.001; **** *p* < 0.0001.


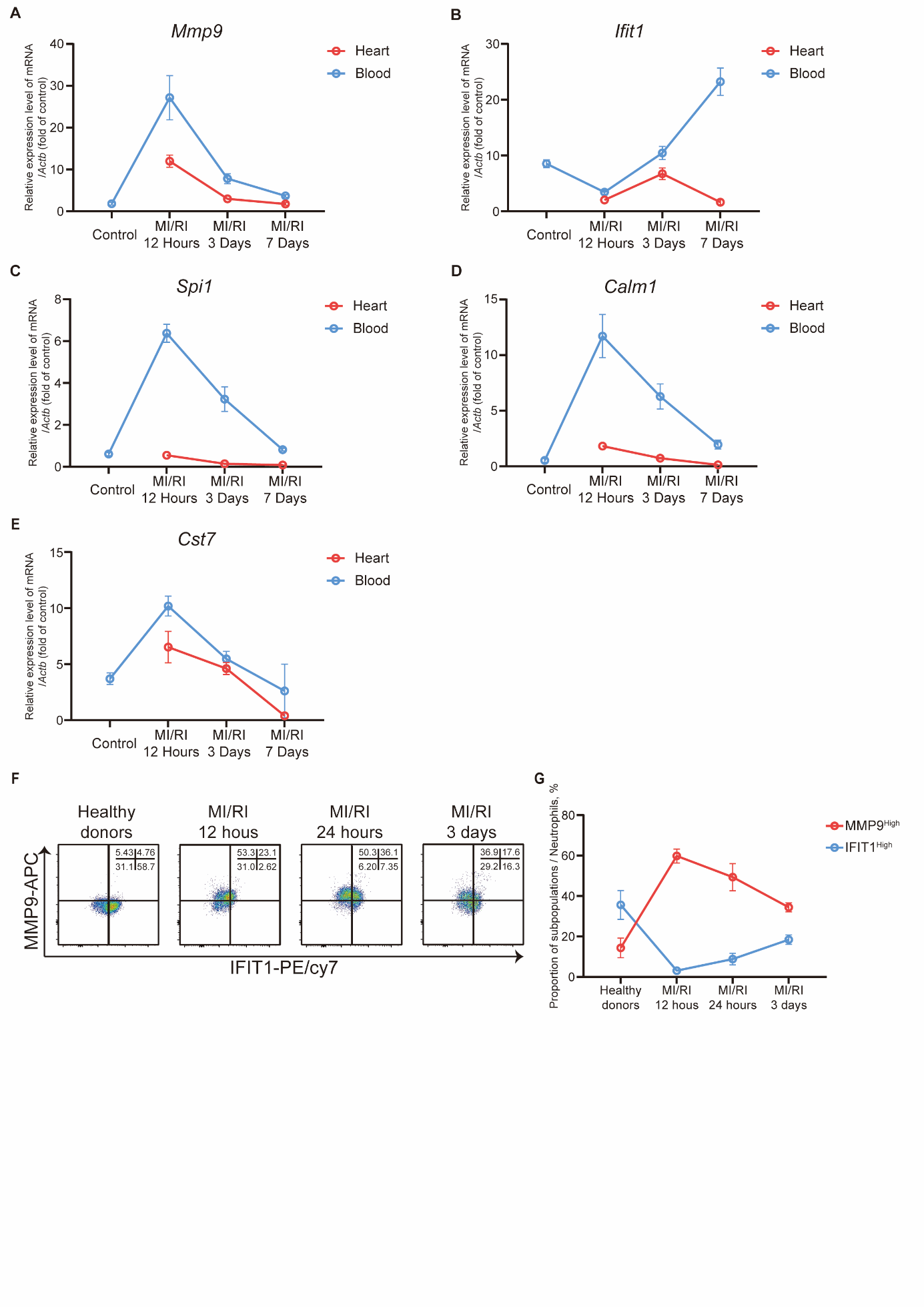


**Figure S7. Time-course study of neutrophil clustering in the mouse model** **and human blood.** **(A-E)** Relative mRNA levels of *Mmp9*, *Ifit1*, *Spi1*, *Calm1*, and *Cst7* of neutrophils FACs sorted from blood and heart of mice along time after MI/RI (*n*=6 each). **(F)** Representative FACs images of neutrophil clustering of neutrophils from human blood at different time points after MI/RI. **(G)** Proportion of MMP9^High^ neutrophils and IFIT1^High^ neutrophils in neutrophils from human blood at different time points after MI/RI (*n*=5 each). All data was displayed as mean ± SEM.


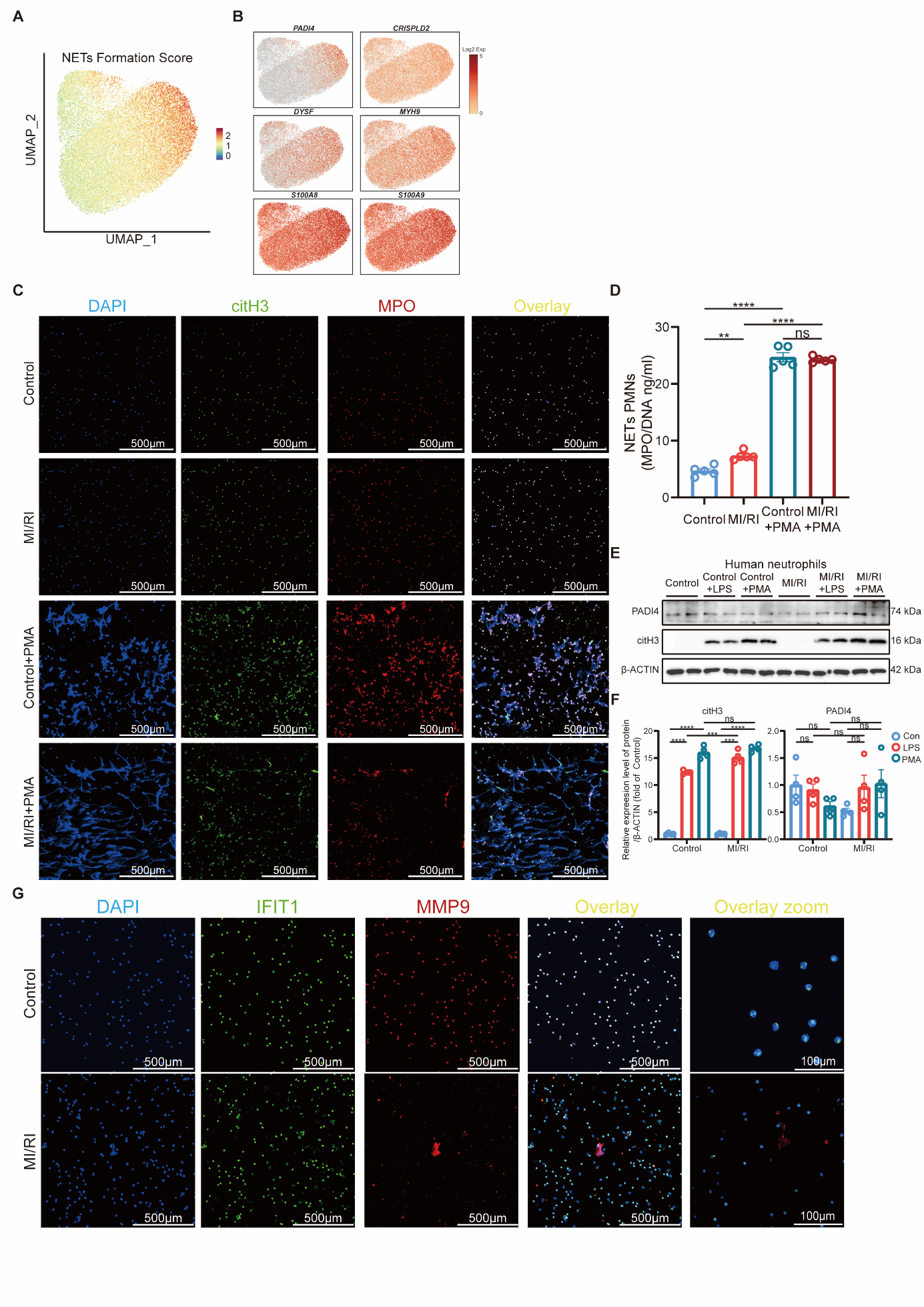


**Figure S8. MMP9^High^ neutrophils account for NET formation. (A)** UMAP plot of NET formation scores. **(B)** UMAP representation of gene expression of marker gene for NET formation (*PADI4*, *CRISPLD2*, *DYSF*, *MYH9*, *S100A8*, and *S100A9*). **(C)** Representative fluorescence images of NETs stained for DNA (DAPI, blue), citH3 (citH3, green), and myeloperoxidase (MPO, red) of human neutrophils, *100, scale bar 500 μm. **(D)** The concentrations of MPO/DNA-NETs (one-way ANOVA test with Tukey's multiple comparisons test) in the neutrophil culture supernatants (*n*=5). **(E-F)** Relative protein levels of PADI4 and citH3 (one-way ANOVA test with Tukey's multiple comparisons test for both) of human neutrophils (*n*=4 each). **(G)** Representative fluorescence images of human neutrophils stained for DAPI (blue), IFIT1 (green), and MMP9 (red) of human neutrophils, *100, scale bar 500 μm; *400, scale bar 100 μm. All data was displayed as mean ± SEM. NS, not significant, *p* > 0.05; ** *p* < 0.01; *** *p* < 0.001; **** *p* < 0.0001.


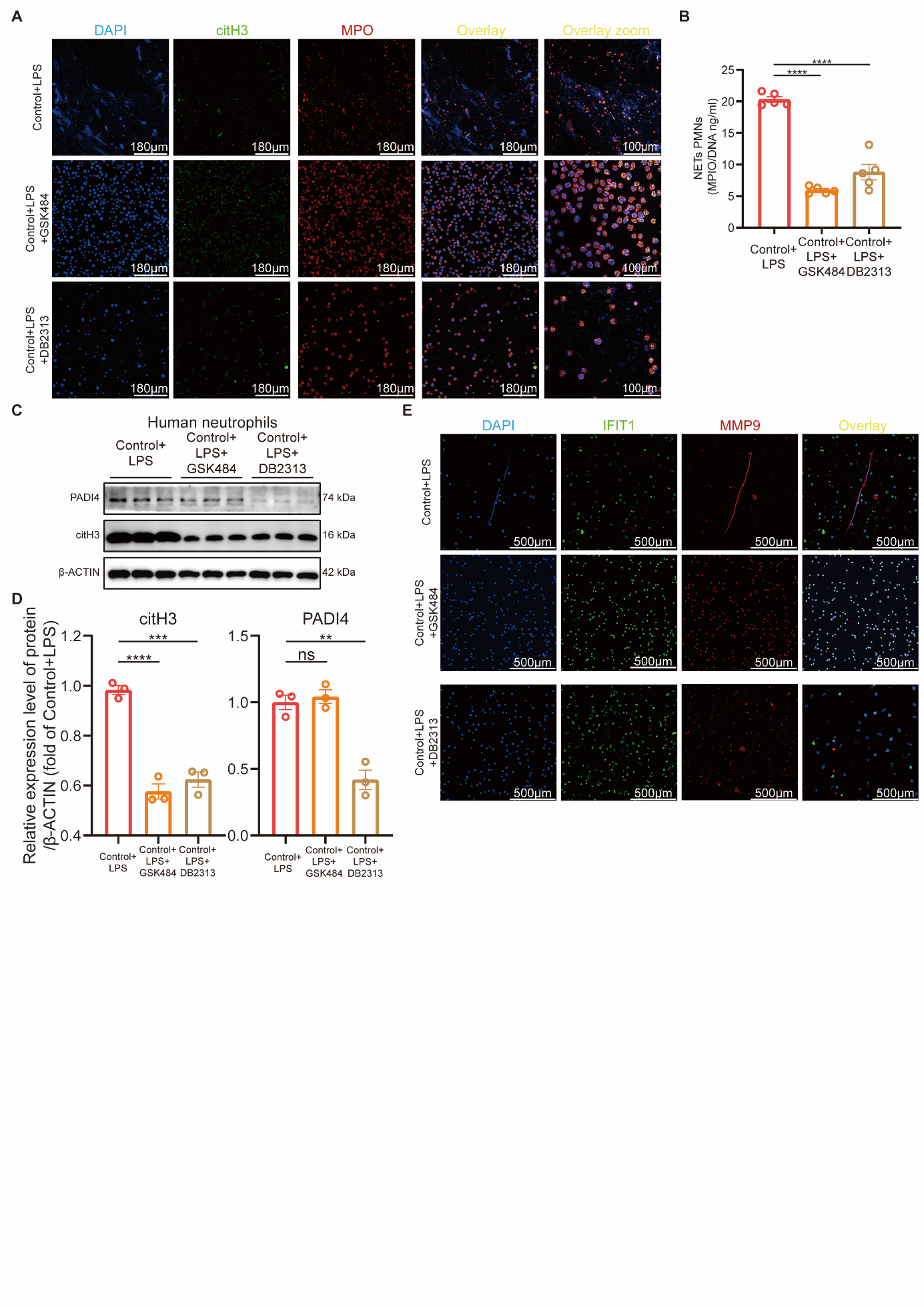


**Figure S9. GSK484 and DB2313 can decrease NET formation *in vitro*. (A)** Representative fluorescence images of NETs stained for DNA (DAPI, blue), citH3 (citH3, green), and myeloperoxidase (MPO, red) of human neutrophils, *200, scale bar 180 μm; *400, scale bar 100 μm. **(B)** The concentrations of MPO/DNA-NETs (one-way ANOVA test with Tukey's multiple comparisons test) in the neutrophil culture supernatants (*n*=5). **(C-D)** Relative protein levels of PADI4 and citH3 (one-way ANOVA test with Tukey's multiple comparisons test for both) of human neutrophils (*n*=3 each). **(E)** Representative fluorescence images of human neutrophils stained for DAPI (blue), IFIT1 (green), and MMP9 (red) of human neutrophils, *100, scale bar 500 μm. All data was displayed as mean ± SEM. NS, not significant, *p* > 0.05; ** *p* < 0.01; *** *p* < 0.001; **** *p* < 0.0001.


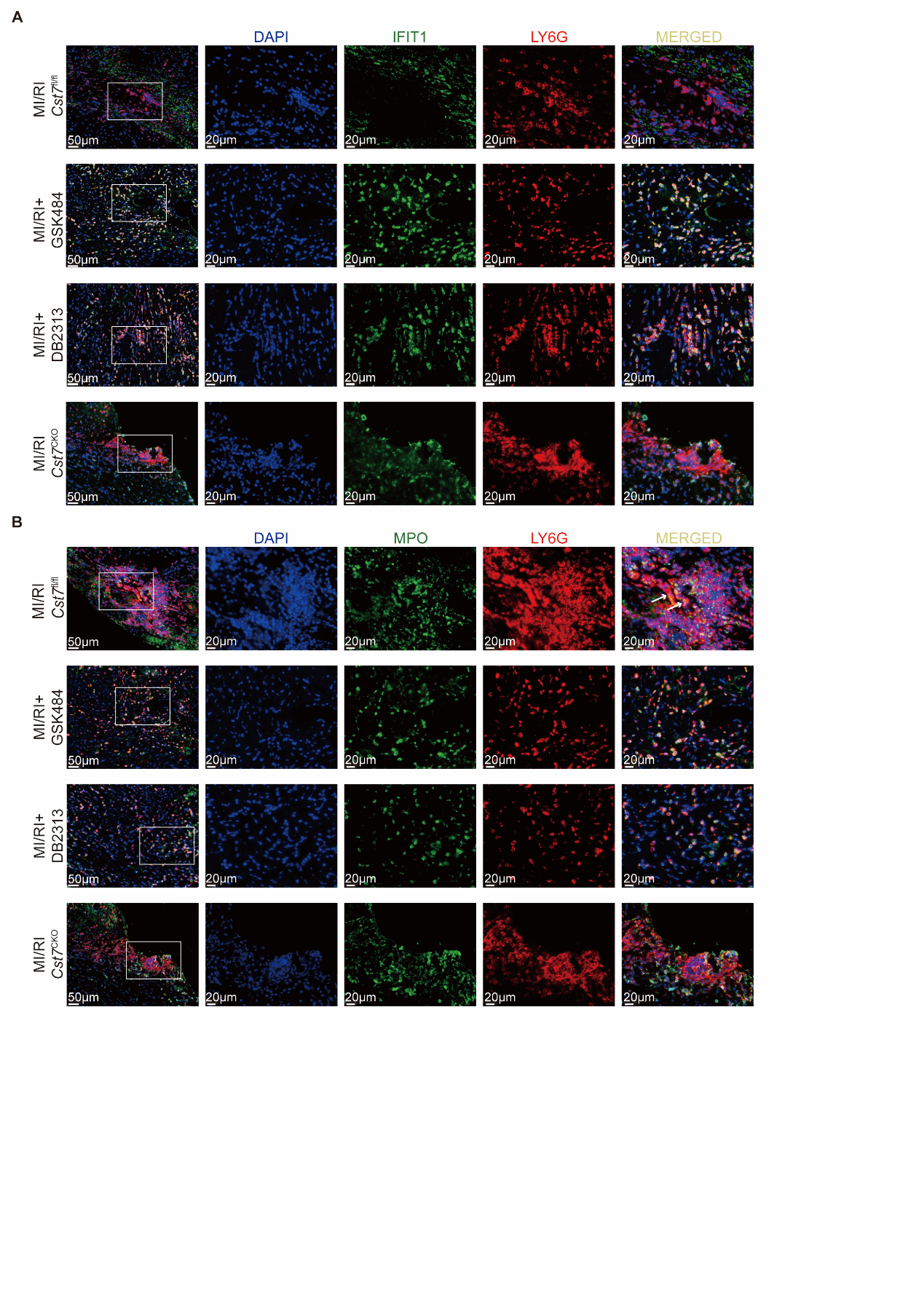


**Figure S10. GSK484, DB2313, or *Cst7*^CKO^ can decrease NET formation and proportion of MMP9^High^ neutrophils *in vivo*. (A)** Representative fluorescence images of neutrophil infiltrated in MI/RI tissue sections stained for DAPI (blue), IFIT1 (green), Ly6G (red), *200, scale bar 50 μm; *400, scale bar 20 μm. **(B)** Representative fluorescence images of NET formation in MI/RI tissue sections stained for DAPI (blue), MPO (green), Ly6G (red), *200, scale bar 50 μm; *400, scale bar 20 μm.


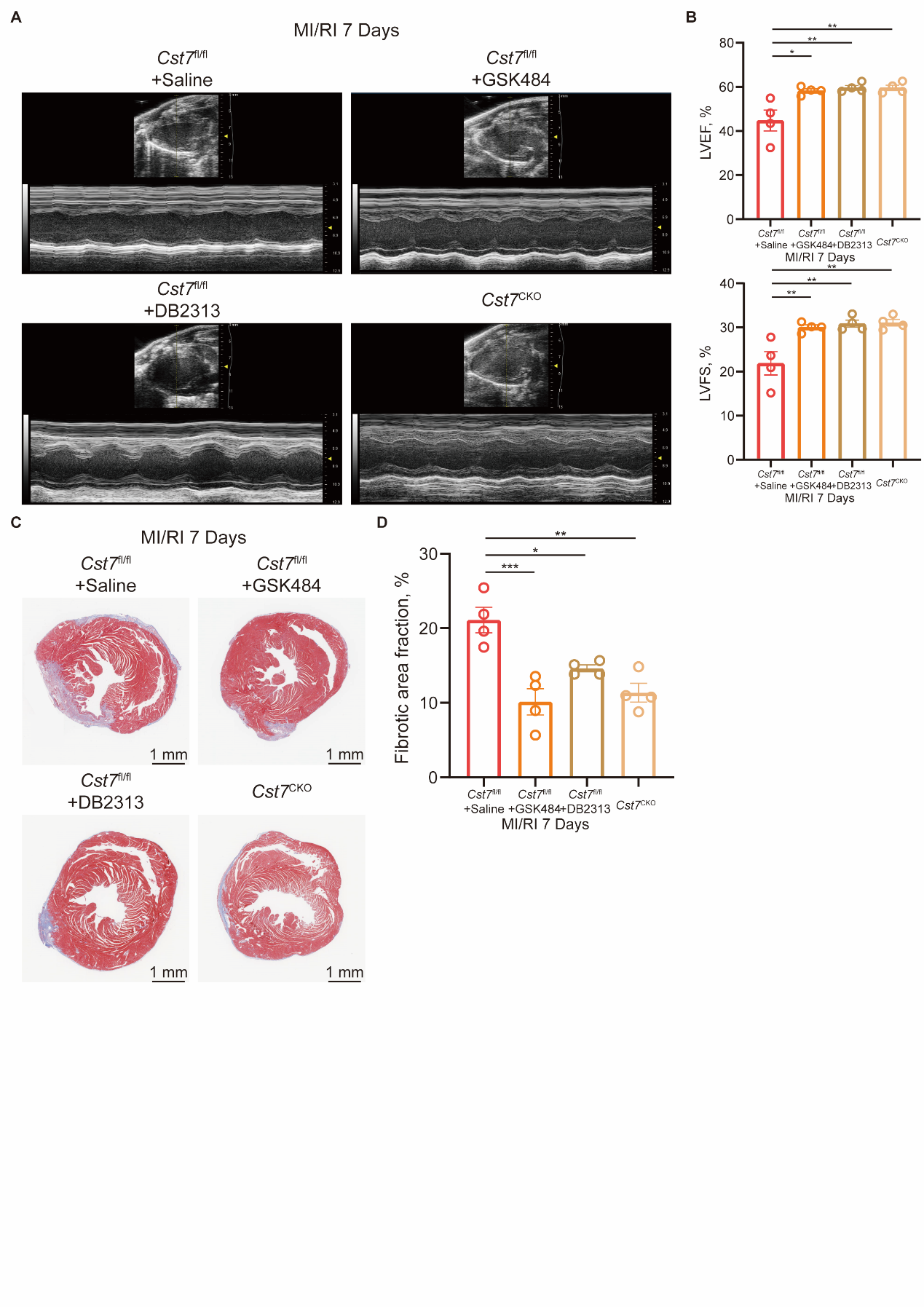


**Figure S11. GSK484, DB2313, or *Cst7*^CKO^ can reduce adverse tissue remodeling 7 days after MI/RI. (A)** Representative images of echocardiography of the mice 7 days after MI/RI. **(B)** LVEF (top) and LVFS (bottom) were compared (*n*=4 and one-way ANOVA test with Tukey's multiple comparisons test for all). **(C)** Representative images of Masson’s trichrome staining (scale bar, 1 mm). **(D)** The fibrotic area fraction was compared was assessed by Masson's trichrome staining (*n*=4 and one-way ANOVA test with Tukey's multiple comparisons test for all). All data was displayed as mean ± SEM. * *p* < 0.05; ** *p* < 0.01; *** *p* < 0.001.


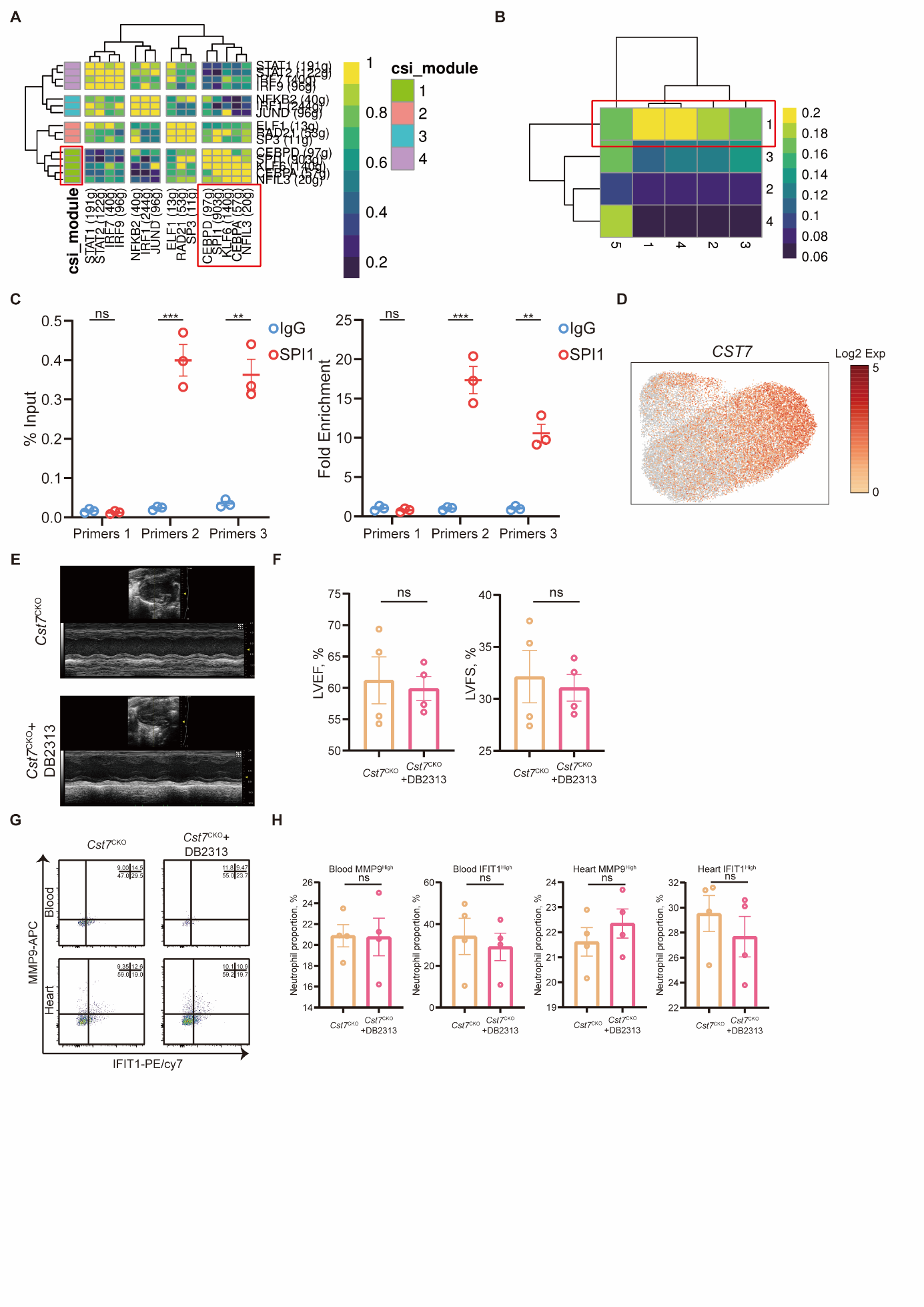


**Figure S12. DB2313 failed to further ameliorate MI/RI and reduce the proportion of MMP9^High^ neutrophils in *Cst7*^CKO^ mice.** **(A)** Heatmap of connection specificity index (CSI) according to modules composed of connected regulons. **(B)** Heatmap of activity of CSI modules according to 5 clusters. **(C)** ChIP-qPCR results of SPI1 on *CST7* promotor, including % Input (left) and Fold Enrichment (right, *n*=3, unpaired *t* test for all). **(D)** UMAP plot of *CST7* expression level of each cluster. **(E)** Representative images of echocardiography of the *Cst7*^CKO^ mice undergoing MI/RI with saline or DB2313 treatment. **(F)** LVEF (left, unpaired *t* test) and LVFS (right, unpaired *t* test) of *Cst7*^CKO^ + MI/RI + vehicle mice (*n*=4) and *Cst7*^CKO^ + MI/RI + DB2313 mice (*n*=4). **(G)** Representative FACs images of neutrophil clustering in blood and heart of *Cst7*^CKO^ + MI/RI + vehicle mice and *Cst7*^CKO^ + MI/RI + DB2313 mice. **(H)** Proportion of MMP9^High^ neutrophils (leftmost, unpaired *t* test) and IFIT1^High^ neutrophils (left middle, unpaired *t* test) in blood of *Cst7*^CKO^ + MI/RI + vehicle mice (*n*=4) and *Cst7*^CKO^ + MI/RI + DB2313 mice (*n*=4). Proportion of MMP9^High^ neutrophils (right middle, unpaired *t* test) and IFIT1^High^ neutrophils (rightmost, unpaired *t* test) in heart of *Cst7*^CKO^ + MI/RI + vehicle mice (*n*=4) and *Cst7*^CKO^ + MI/RI + DB2313 mice (*n*=4). All data was displayed as mean ± SEM. NS, not significant, *p* > 0.05; ** *p* < 0.01; *** *p* < 0.001.

**Table S1 and Table S7**

| Characteristics | MI/RI (*n*=3) | Control (*n*=3) |
| --- | --- | --- |
| Age | 59.00±3.61 | 67.67±9.29 |
| Sex, male (%) | 3 (100.0) | 1 (33.3) |
| Hypertension (%) | 2 (66.7) | 1 (33.3) |
| Hyperlipidemia (%) | 1 (33.3) | 0 (0.0) |
| Diabetes (%) | 0 (0.0) | 0 (0.0) |
| Smoke (%) | 2 (66.7) | 0 (0.0) |
| Time from chest pain onset to balloon inflation (hours) | 3.67±0.88 |  |
| STEMI (%) | 3 (100) |  |
| Ejection fraction (%) | 55.33±6.43 | 67.33±0.58 |
| cTnT peak (ng/mL) | 5.61±2.96 | 0.00±0.01 |
| CK-MB peak (U/L) | 214.00±103.76 | 18.67±2.52 |
| Pro-BNP peak (pg/mL) | 2338.00±1782.96 | 100.87±108.65 |
| LDH peak (U/L) | 548.00±473.86 | 192.00±19.97 |
| WBC count (10^9^/L) | 11.00±1.88 | 6.81±2.05 |
| Neutrophil percentage (%) | 71.20±11.84 | 58.40±12.66 |
| Lymphocyte percentage (%) | 19.43±10.20 | 30.23±11.35 |
| NLR% | 4.49±2.41 | 2.22±1.15 |
| Neutrophil count (10^9^/L) | 7.90±2.19 | 4.13±1.82 |
| Lymphocyte count (10^9^/L) | 2.07±0.80 | 1.93±0.50 |
| NLR | 4.42±2.32 | 2.25±1.16 |

**Table S1. Clinical characteristics of 3 patients with MI/RI and 3 healthy donors whose peripheral blood was used for scRNA-seq.**

Data presented as mean ± standard deviation or number (percentage). Statistical analysis was described in methods. STEMI, ST-elevated myocardial infarction; cTnT, cardiac troponin T; CK-MB, creatine kinase MB isoenzyme; pro-BNP, processor brain natriuretic peptide; LDH, lactic dehydrogenase; WBC, white blood cell; NLR, neutrophil-lymphocyte ratio.

| Characteristics | MI/RI (*n*=31) | Control (*n*=22) | *P* |
| --- | --- | --- | --- |
| Age | 62.65±12.87 | 57.95±12.63 | 0.200 |
| Sex, male (%) | 28 (90.3) | 11 (50.0) | 0.001 |
| Hypertension (%) | 16 (51.6) | 11 (52.4) | 0.957 |
| Hyperlipidemia (%) | 5 (6.1) | 4 (19.0) | 0.785 |
| Diabetes (%) | 13 (41.9) | 3 (14.3) | 0.034 |
| Smoke (%) | 9 (29.0) | 3 (4.3) | 0.216 |
| COPD (%) | 1 (3.2) | 0 (0) | 0.406 |
| Strock (%) | 1 (3.2) | 1 (4.8) | 0.777 |
| Family history of coronary heart disease (%) | 1 (3.2) | 0 (0) | 0.406 |
| Time from chest pain onset to balloon inflation (hours) | 7.097±0.69 |  |  |
| STEMI (%) | 26 (83.9) |  |  |
| Ejection fraction (%) | 53.55±8.68 | 65.73±4.65 | 0.000 |
| cTnT peak (ng/mL) | 3.99±3.52 | 0.20±0.65 | 0.000 |
| CK-MB peak (U/L) | 179.39±167.06 | 18.98±24.74 | 0.000 |
| Pro-BNP peak (pg/mL) | 2049.91±2313.16 | 107.61±112.41 | 0.000 |
| LDH peak (U/L) | 700.65±520.35 | 166.27±59.22 | 0.000 |
| Platelet (10^9^/L) | 211.2±61.85 | 224.14±37.79 | 0.388 |
| WBC count (10^9^/L) | 10.98±3.03 | 6.41±1.49 | 0.000 |
| Neutrophil percentage (%) | 75.30±7.50 | 55.29±10.21 | 0.000 |
| Lymphocyte percentage (%) | 15.85±6.33 | 34.10±9.53 | 0.000 |
| NLR% | 6.06±3.90 | 1.87±0.97 | 0.000 |
| Neutrophil count (10^9^/L) | 8.39±2.97 | 3.62±1.40 | 0.000 |
| Lymphocyte count (10^9^/L) | 1.66±0.68 | 2.00±0.57 | 0.630 |
| NLR | 6.04±3.90 | 2.07±1.21 | 0.000 |
| Creatinine (umol/L) | 79.13±14.09 | 75.46±18.57 | 0.416 |
| D-Dimer (mg/L) | 0.69±0.85 | 0.16±0.16 | 0.002 |
| Hs-CRP (mg/L) | 17.29±28.15 | 1.55±2.34 | 0.004 |

**Table S7. Clinical characteristics of 31 patients with MI/RI and 22 healthy donors whose** **peripheral blood was used for verification.**

Data presented as mean ± standard deviation or number (percentage). Statistical analysis was described in methods. STEMI, ST-elevated myocardial infarction; cTnT, cardiac troponin T; CK-MB, creatine kinase MB isoenzyme; pro-BNP, processor brain natriuretic peptide; LDH, lactic dehydrogenase; WBC, white blood cell; NLR, neutrophil-lymphocyte ratio; Hs-CRP, hypersensitive-c-reactive-protein.
